# Supplementary material for: Mature tertiary lymphoid structures evoke intra-tumoral T and B cell responses via progenitor exhausted CD4+ T cells in head and neck cancer
Source: Nat Commun. 2025 May 7;16:4228. doi: 10.1038/s41467-025-59341-w (PMC12059173; doi:10.1038/s41467-025-59341-w)
Supplement: Supplementary file 1 — Supplementary Information [file 41467_2025_59341_MOESM1_ESM.pdf]

Mature tertiary lymphoid structures evoke intra-tumoral T and B cell responses via progenitor exhausted CD4⁺ T cells in head and neck cancer

Hao Li[1,2,#], Meng-Jie Zhang[1,#], Boxin Zhang[1], Wen-Ping Lin[1], Shu-Jin Li[1], Dian Xiong[1], Qing Wang[1], Wen-Da Wang[1], Qi-Chao Yang[1], Cong-Fa Huang[1], Wei-Wei Deng[1,2,*], Zhi-Jun Sun[1,2*]

[1]State Key Laboratory of Oral & Maxillofacial Reconstruction and Regeneration, Key Laboratory of Oral Biomedicine Ministry of Education, Hubei Key Laboratory of Stomatology, School & Hospital of Stomatology, Frontier Science Center for Immunology and Metabolism, Taikang Center for Life and Medical Sciences, Wuhan University, Wuhan, China.

[2]Department of Oral Maxillofacial-Head Neck Oncology, School & Hospital of Stomatology, Wuhan University, Wuhan, China.

[#]These authors contributed equally to this work.

[*]Email: sunzj@whu.edu.cn, dww@whu.edu.cn

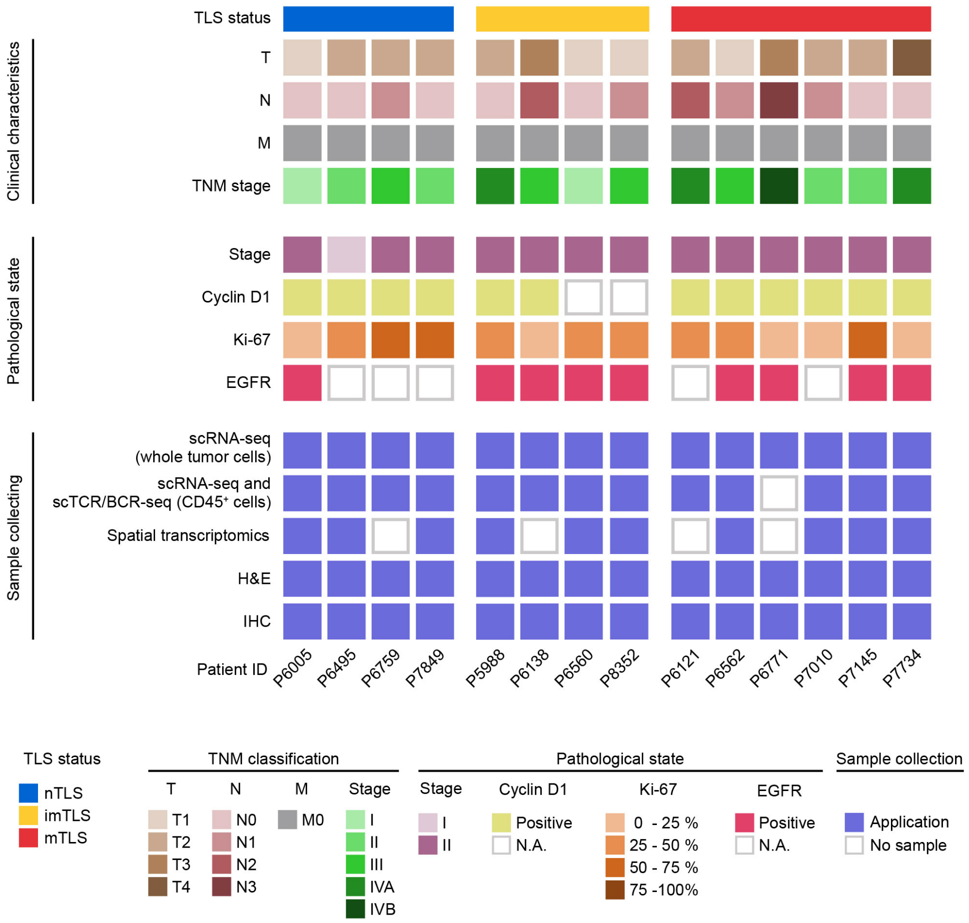

Supplementary Fig. 1 | Clinicopathological parameter analysis of HNSCC patients. Clinical metadata and analyses performed for each patient with different status of TLS. Patient data include TLS status, patient age, TNM classification, pathological state. The data collection methods included scRNA-seq for whole tumor cells, paired scRNA-seq and scTCR/BCR-seq for intra-tumoral CD45$^+$ cells, spatial transcriptomics, H&E and IHC.

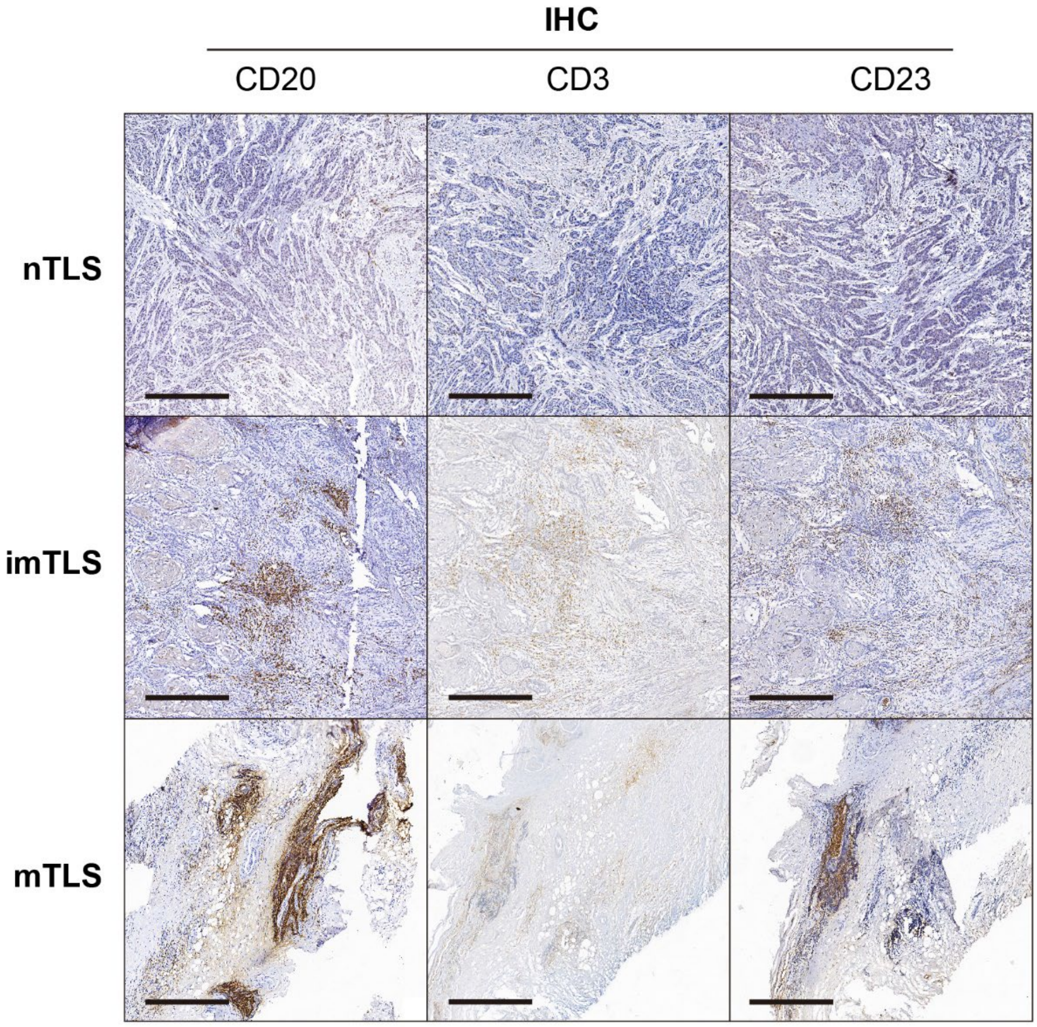

Supplementary Fig. 2 | Assessment of the presence and status of TLS in HNSCC. This panel shows representative examples of non-TLS (nTLS), immature TLS (imTLS) and mature TLS (mTLS) observed in HNSCC samples. mTLS are defined by the presence of a network of CD23-positive cells and accumulation of CD20-positive B cells and CD3-positive T cells on IHC. imTLS are distinguished by an accumulation of CD20-positive B cells and CD3-positive T cells, but lack the distinctive CD23-positive cell network observed under IHC. nTLS, conversely, are marked by the absence of both CD20-positive B cells and CD3-positive T cells. nTLS and imTLS were repeated 4 times independently with similar results, mTLS were repeated 6 times independently with similar results. Scale bars = 500 μm.

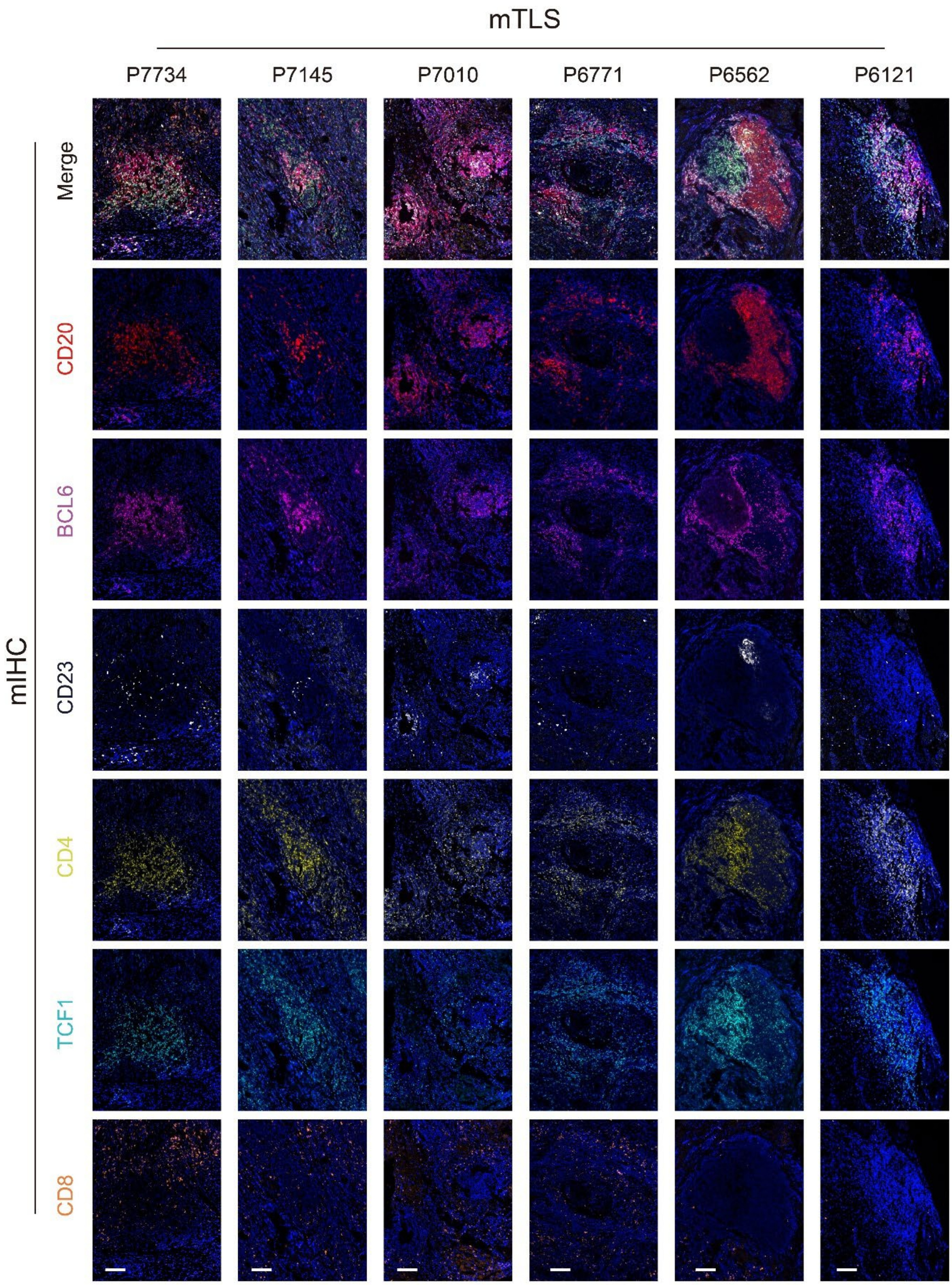

Supplementary Fig. 3 | Assessment of the presence and status of mTLS by mIHC. Regions with a high density of GC B cells (CD20, BCL6) and T cells (CD4, CD8, TCF1), and follicular dendritic cells (FDCs) (CD23). mTLS were repeated 6 times independently with similar results. Scale bars = 100 µm. Images of a mIHC-stained tumor of mTLS showed aggregation of $CD20^{+}BCL6^{+}$ B cells surround by $CD4^{+}TCF1^{+}$ T cells and $CD8^{+}$ T cells. $CD23^{+}$ FDCs were distributed in networks or scattered lymphoid aggregates.

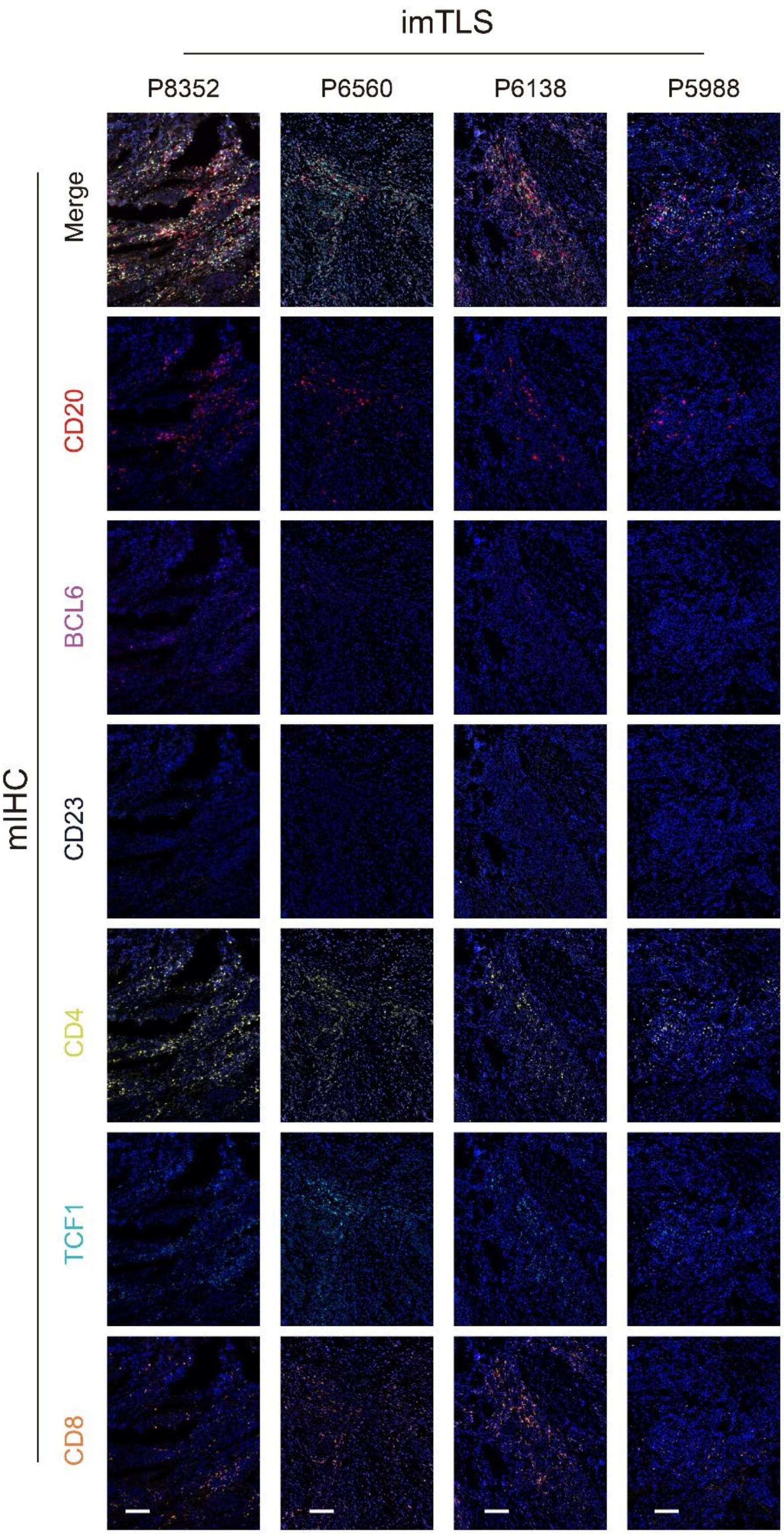

Supplementary Fig. 4 | Assessment of the presence and status of imTLS by mIHC. Regions with a high density of GC B cells (CD20, BCL6) and T cells (CD4, CD8, TCF1), and follicular dendritic cells (FDCs) (CD23). imTLS were repeated 4 times independently with similar results. Scale bars = 100 μm. Images of a mIHC-stained tumor of imTLS showed aggregation of $CD20^{+}BCL6^{+}$ B cells surround by $CD4^{+}TCF1^{+}$ T cells and $CD8^{+}$ T cells. There was no distribution of $CD23^{+}$ FDCs in lymphoid aggregates.

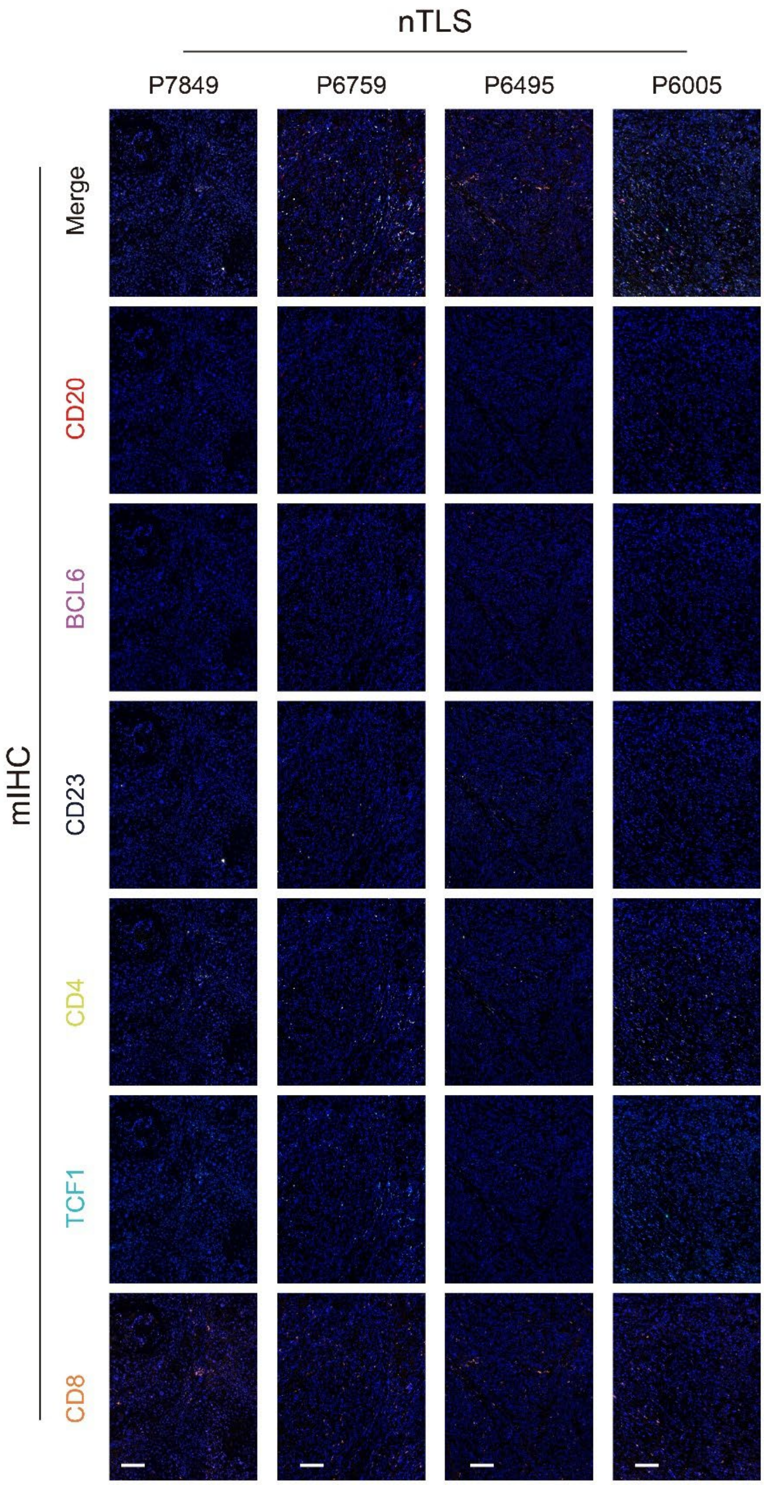

Supplementary Fig. 5 | Assessment of the presence and status of imTLS by nIHC. Regions with a high density of GC B cells (CD20, BCL6) and T cells (CD4, CD8, TCF1), and follicular dendritic cells (FDCs) (CD23). nTLS were repeated 4 times independently with similar results. Scale bars = 100 μm. Images of a mIHC-stained tumor of nTLS showed a scattered distribution of B cells and T cells. There was no distribution of CD23^{+} FDCS in lymphoid aggregates.

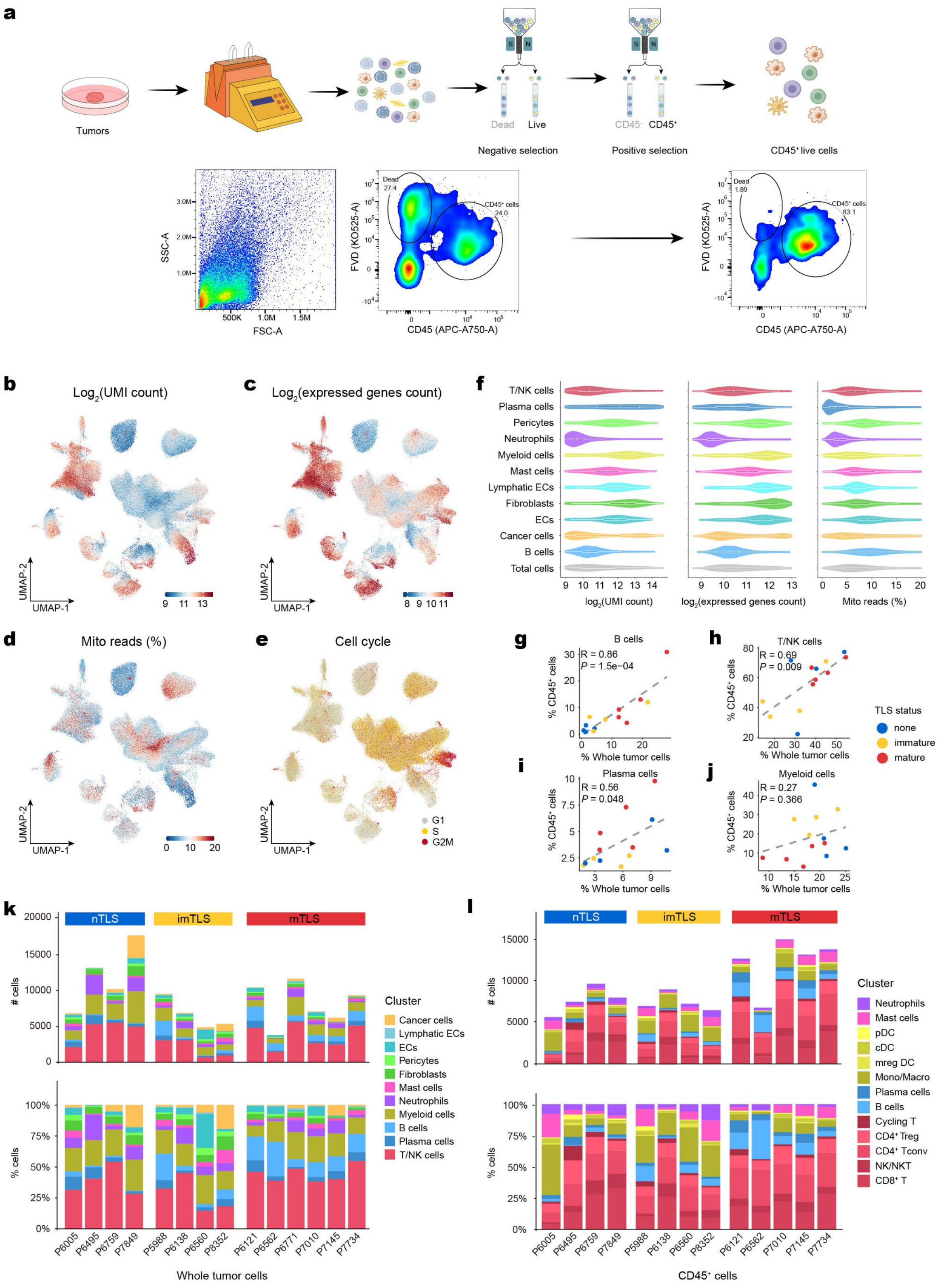

Supplementary Fig. 6 | Quality control and cell type abundance profiled by scRNA-seq of HNSCC. a Schematic diagram of the collection process of $CD45^+$ cells in tumor, including sample collection, magnetic bead sorting and flow cytometry detection. **b-e** UMAP plots of cells profiled by scRNA-seq colored by different quality

metrics: log_2 (UMI counts), log_2 (expressed gene count), fraction of mitochondrial reads, and cell cycle phase. **f** Distribution of quality metrics for each annotated cell type. **g-j** Correlation between the fraction of whole tumor cells and the fraction of $CD45^+$ cell in tumor in B cells (**g**), T/NK cells (**h**), plasma cells (**i**) and myeloid cells (**j**). **k, l** Cell counts and cell type compositions of whole tumor cell (**k**) and sorted intratumoral $CD45^+$ cells (**l**) based on scRNA-seq, separated by HNSCC patients, ranked by nTLS, imTLS, mTLS respectively.

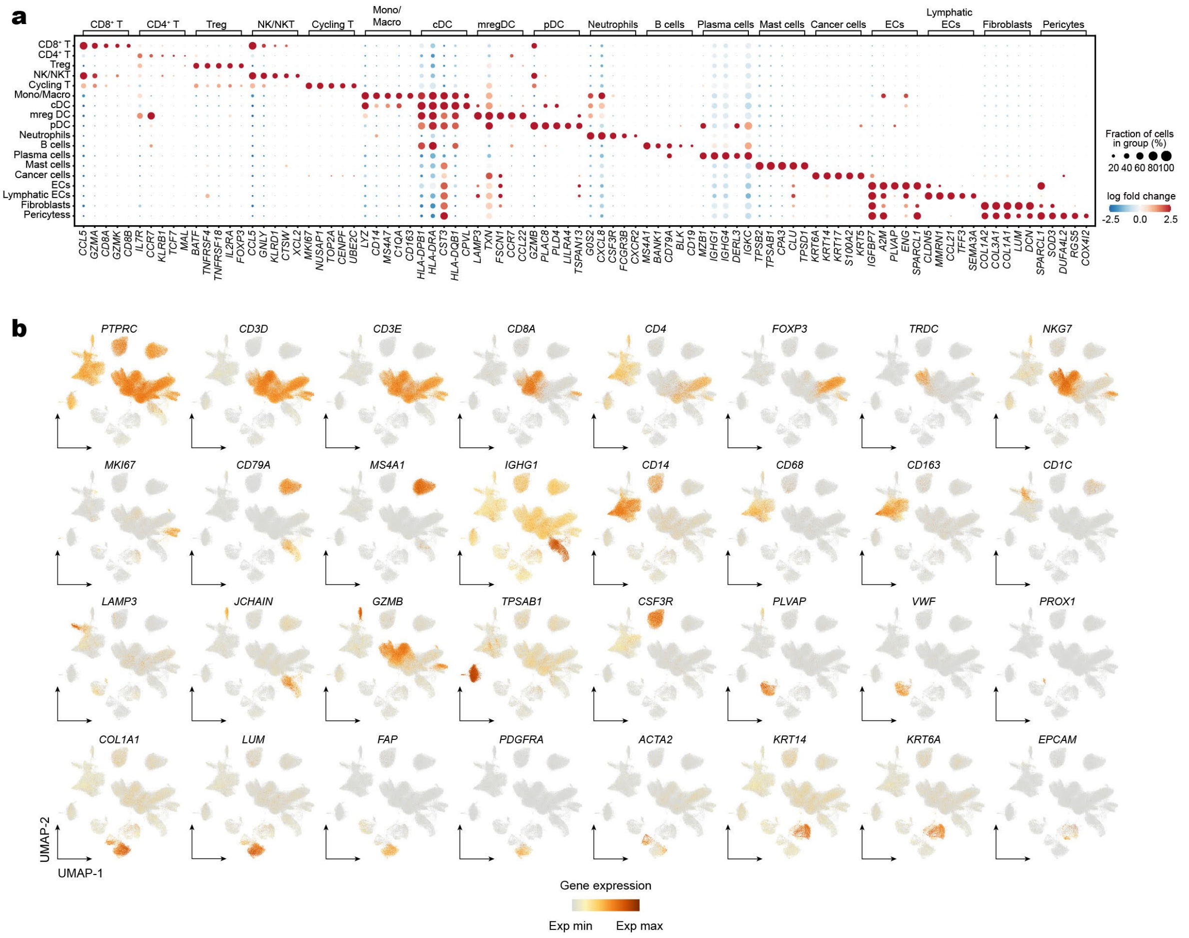

Supplementary Fig. 7 | Marker gene expression of various cell phenotypes. a, Dot plot of scaled marker gene expression (averaged per cluster) for coarse-grained T/NK cell subclusters, showing differentially expressed genes in columns and clusters in rows. Genes are grouped by cluster and five genes per cluster that are highlighted. A color gradient represents the mean expression within each of the subcluster of T/NK cells, while the sizes of the depicted points indicate the fraction of cells in the categories expressing a gene. **b,** UMAP plot of normalized expression of specific markers.

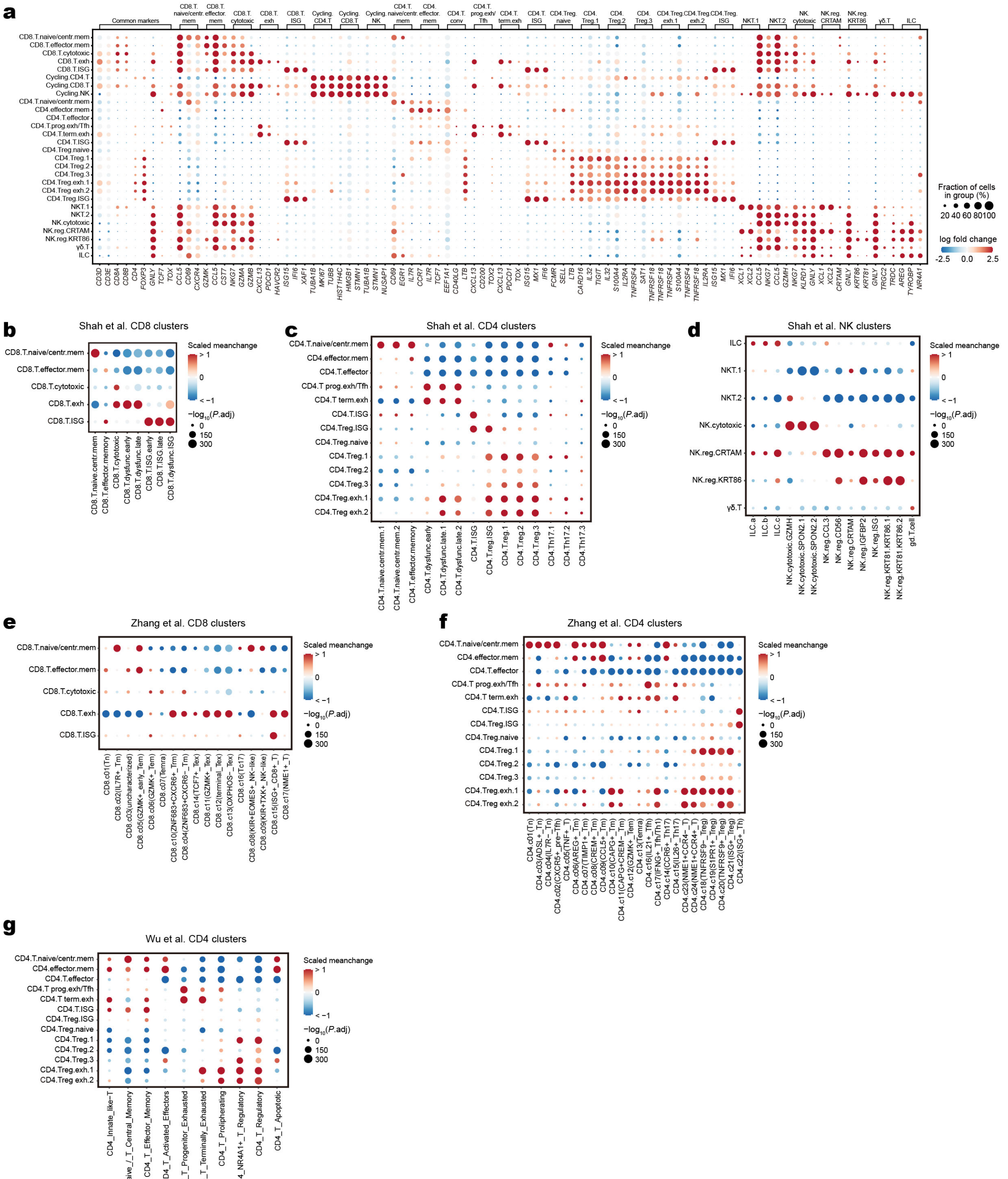

Supplementary Fig. 8 | Marker gene expression of T/NK cell subclusters. a, Dot plot of scaled marker gene expression (averaged per cluster) for coarse-grained T/NK cell subclusters, showing differentially expressed genes in columns and clusters in rows. Genes are grouped by cluster and three genes per cluster that are highlighted. A color gradient represents the mean expression within each of the subcluster of T/NK cells, while the sizes of the

depicted point indicate the fraction of cells in the categories expressing a gene. **b-g,** Dot plot showing comparison of T/NK cell subclusters (this study) and published T/NK cell subclusters using AUCell algorithm to verify the cell annotation. A color gradient, transitioning from red (representing enrichment) to blue (representing depletion), encodes the Z-scaled scores, while the sizes of the depicted points are governed by the Benjamini-Hochberg-adjusted -$\log_{10}$(*P* values).

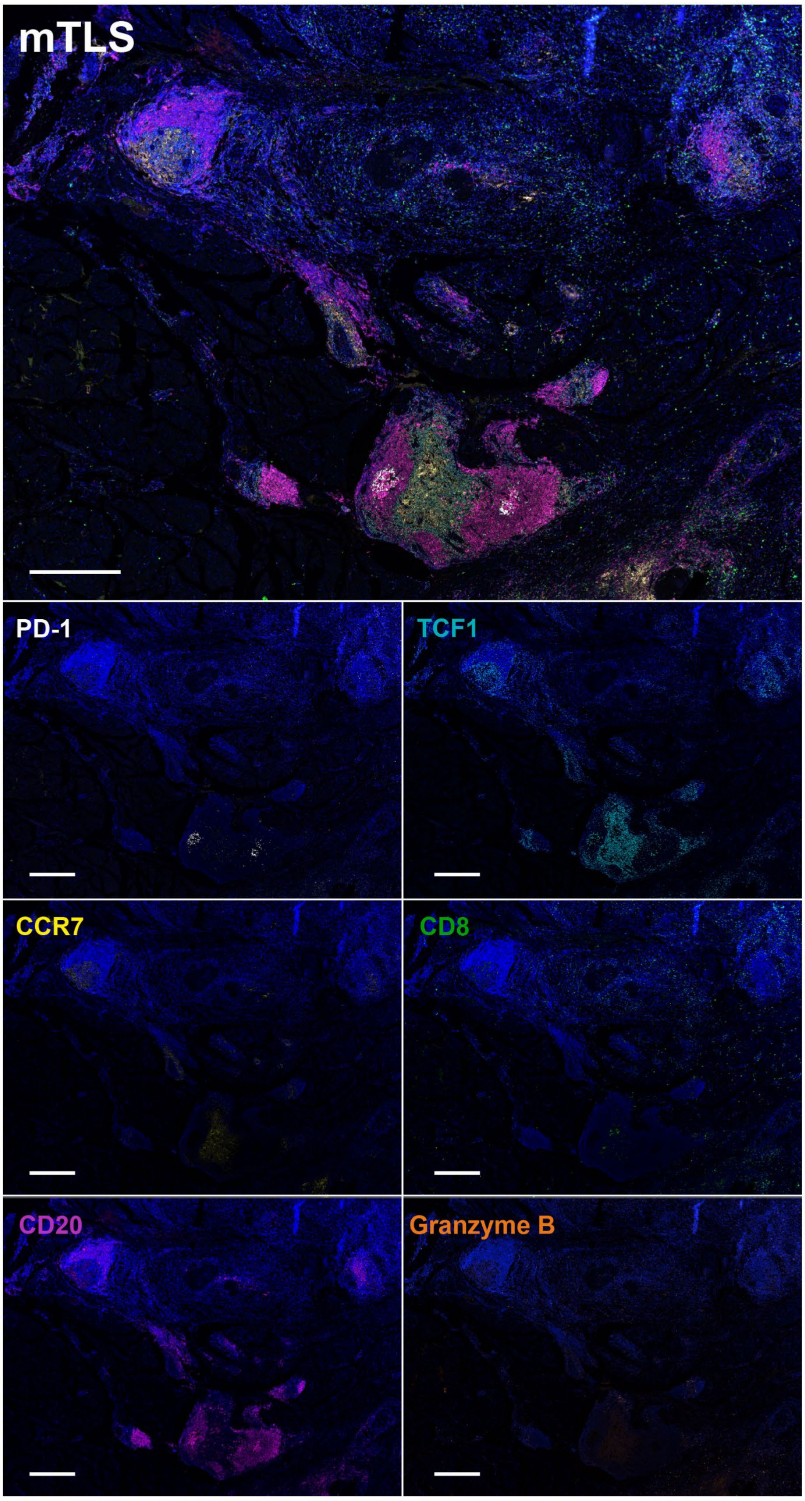

Supplementary Fig. 9 | Representative images of a mIHC-stained different subclusters of CD8^{+} T cells in HNSCC tumor with mTLS status. Multiplex immunofluorescence assay of CD8 (green), PD-1 (white), TCF1 (cyan), CCR7 (yellow), CD20 (magenta), Granzyme B (orange) and DAPI (blue). nTLS and imTLS were repeated 4 times independently with similar results, mTLS were repeated 6 times independently with similar results. Scale bars = 500 μm.

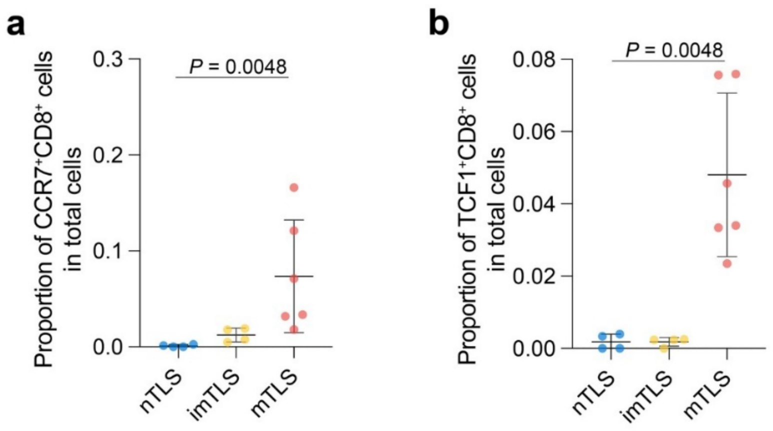

Supplementary Fig. 10 | The statistics of the subclusters of $CD8^+$ T cells in an mIHC-stained HNSCC tumor with mTLS status. Proportion of $CCR7^+CD8^+$ cells (**a**) and $TCF1^+CD8^+$ cells (**b**) in total cells of HNSCC tissue with different TLS status, respectively 14 (n = 4 independent samples nTLS, n = 4 independent samples imTLS, n = 6 independent samples mTLS, one-tailed Mann-Whitney *U*-test; for dot plots: center line, mean; whiskers, standard deviation).

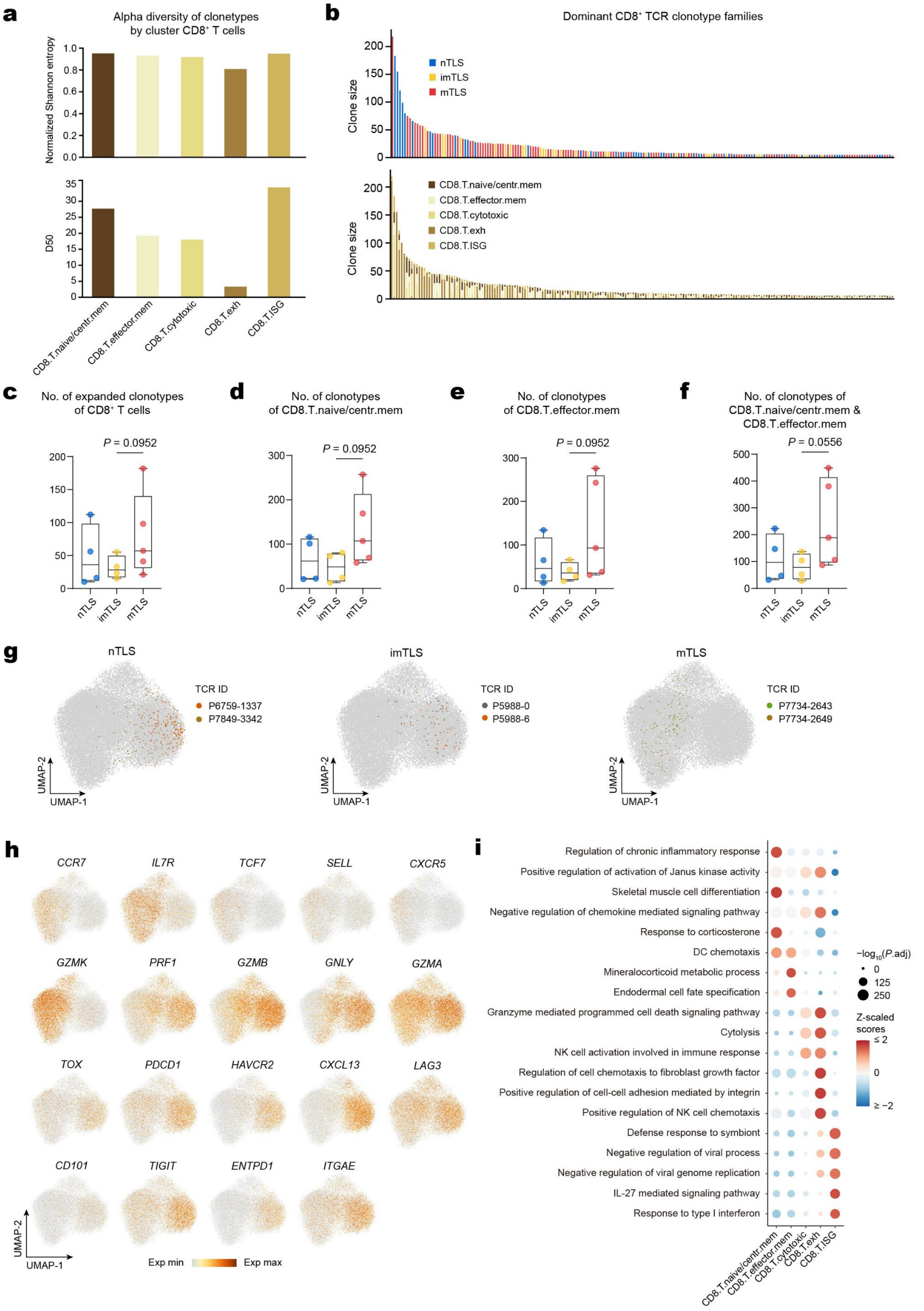

Supplementary Fig. 11 | Basic information of TLS-associated CD8$^+$ T cell clusters in HNSCC. a, Boxplot showing the alpha diversity of clonotypes, including normalized Shannon entropy and D50 index, quantified

across CD8$^+$ T cell clusters. **b,** Cluster distribution of the top 200 CD8$^+$ TCR clonotype families. Colors indicate TLS status or cell subcluster. **c-f,** Boxplot showed the number of expanded clonotypes of total CD8$^+$ T cells (**c**), naive/central memory CD8$^+$ T cells (**d**), effector memory CD8$^+$ T cells (**e**) and stem-like CD8$^+$ T cells (**f**) in different status of TLS (n = 4 independent samples nTLS, n = 4 independent samples imTLS, n = 5 independent samples mTLS, one-tailed Mann-Whitney *U*-test; for box plots: box center line, median; box limits, upper and lower quartiles; box whiskers, maximum and minimum values). **g,** UMAP plot showing the distribution of representative TCR clonotypes in different TLS status. **h,** UMAP plots showing normalized expression profiles of cell-type-specific markers in CD8$^+$ T cell subclusters. **i,** Dot plot showing the analysis of enrichment for the four most significant GO Biological Process terms across CD8$^+$ T cell subclusters. A color gradient, transitioning from red (representing enrichment) to blue (representing depletion), encodes the Z-score normalized enrichment score, while the sizes of the depicted points are governed by the Benjamini-Hochberg-adjusted $-\log_{10}(P$ values), highlighting the statistical significance of observed variations. Benjamini-Hochberg-adjusted *P* values were obtained by one-tailed Wilcoxon rank-sum test.

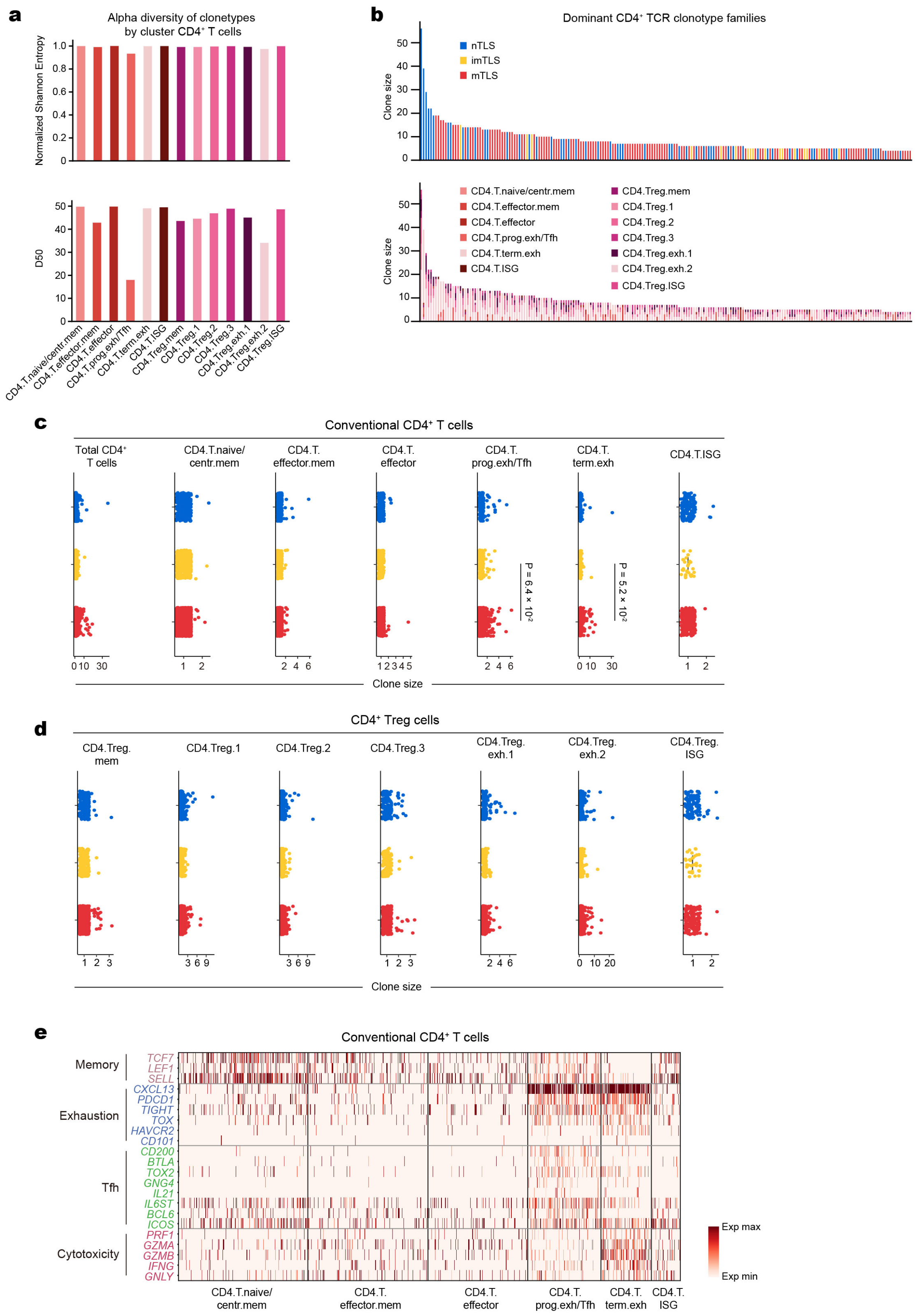

Supplementary Fig. 12 | Basic information of TLS-associated CD4$^+$ T cell clusters in HNSCC. a, Boxplot showing the alpha diversity of clonotypes, including normalized Shannon entropy and D50 index, quantified

across CD4$^+$ T cell clusters. **b,** Cluster distribution of the top 200 CD4$^+$ TCR clonotype families. Colors indicate TLS status or cell subcluster. **c, d,** Clone size in each CD4$^+$ Tconv cell subclusters (**c**) and CD4$^+$ Treg cell (**d**) subclusters separated by TLS status (two-tailed Mann-Whitney *U*-test). **e,** Heatmap plot depicting scaled expression of genes associated with memory, exhaustion, Tfh and cytotoxicity in CD4$^+$ Tconv cells.

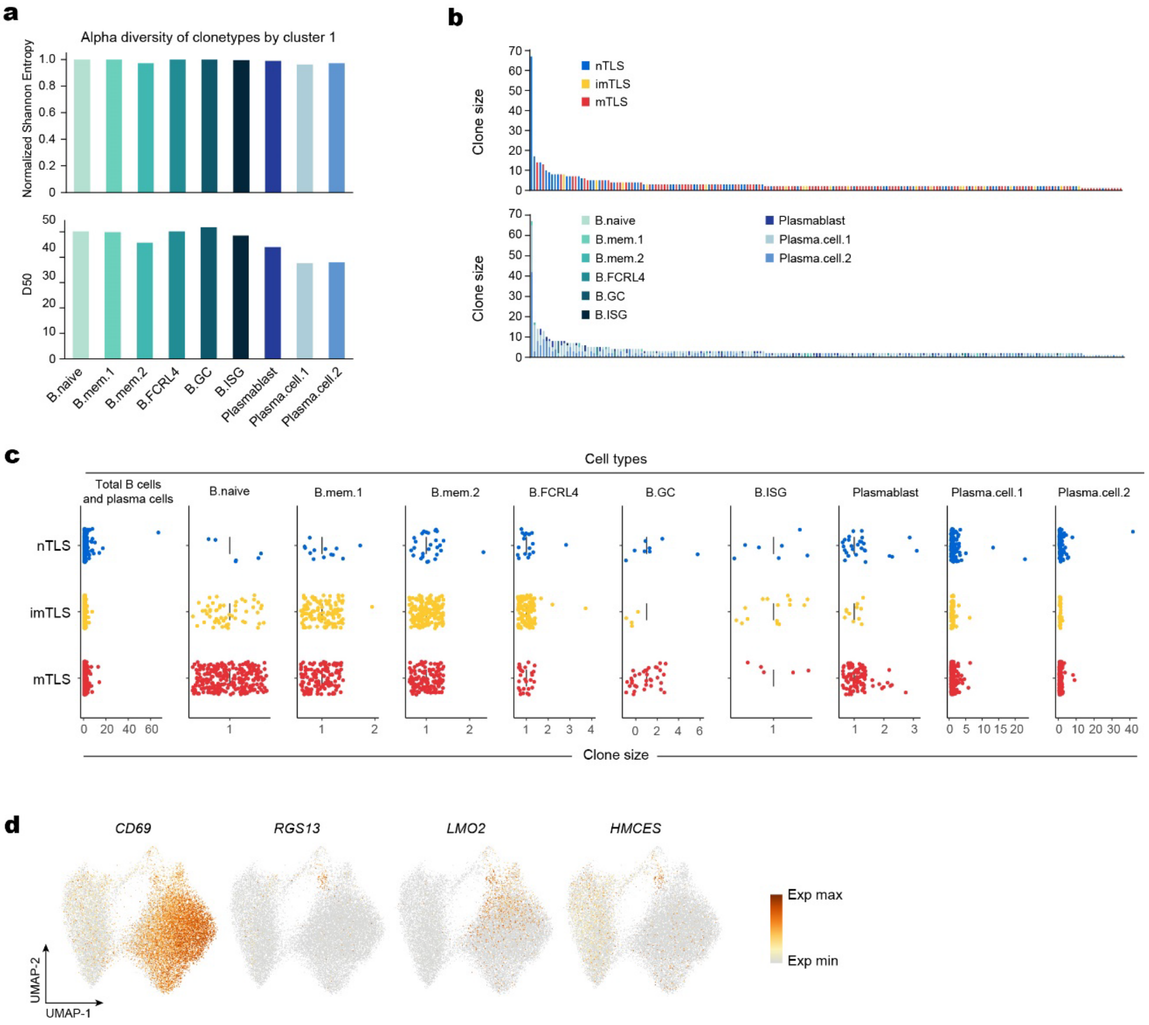

Supplementary Fig. 13 | Basic information of TLS-associated B cell clusters in HNSCC. a, Boxplot showing the alpha diversity of clonotypes, including normalized Shannon entropy and D50 index, quantified across B/plasma cell T cell clusters. **b,** Cluster distribution of the top 200 BCR clonotype families. Colors indicate TLS status or cell subcluster. **c,** Clone size in each B/plasma cell subclusters separated by TLS status (two-tailed Mann-Whitney *U*-test). **d,** UMAP plots showing normalized expression profiles of cell-type-specific markers in B/plasma cell T cell subclusters.

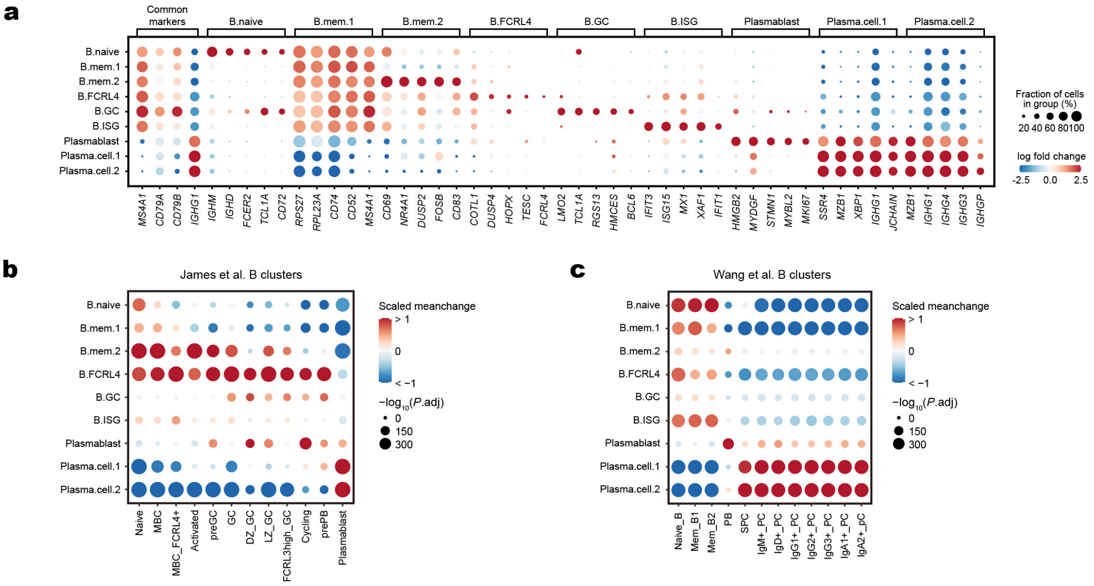

Supplementary Fig. 14 | Marker gene expression of B cell and plasma cell phenotypes. a, Dot plot of scaled marker gene expression (averaged per cluster) for coarse-grained B cell subclusters, showing differentially expressed genes in columns and clusters in rows. Genes are grouped by cluster and three genes per cluster that are highlighted. A color gradient represents the mean expression within each of the subcluster of B cells, while the sizes of the depicted point indicate the fraction of cells in the categories expressing a gene. **b, c**, Dot plot showing comparison of B cell subclusters (this study) and published B/Plasma cell subclusters using AUCell algorithm to verify the cell annotation. A color gradient, transitioning from red (representing high scaled mean change) to blue (representing high scaled mean change), encodes the Z-scaled scores, while the sizes of the depicted points are governed by the Benjamini-Hochberg-adjusted -$\log_{10}$(*P* values).

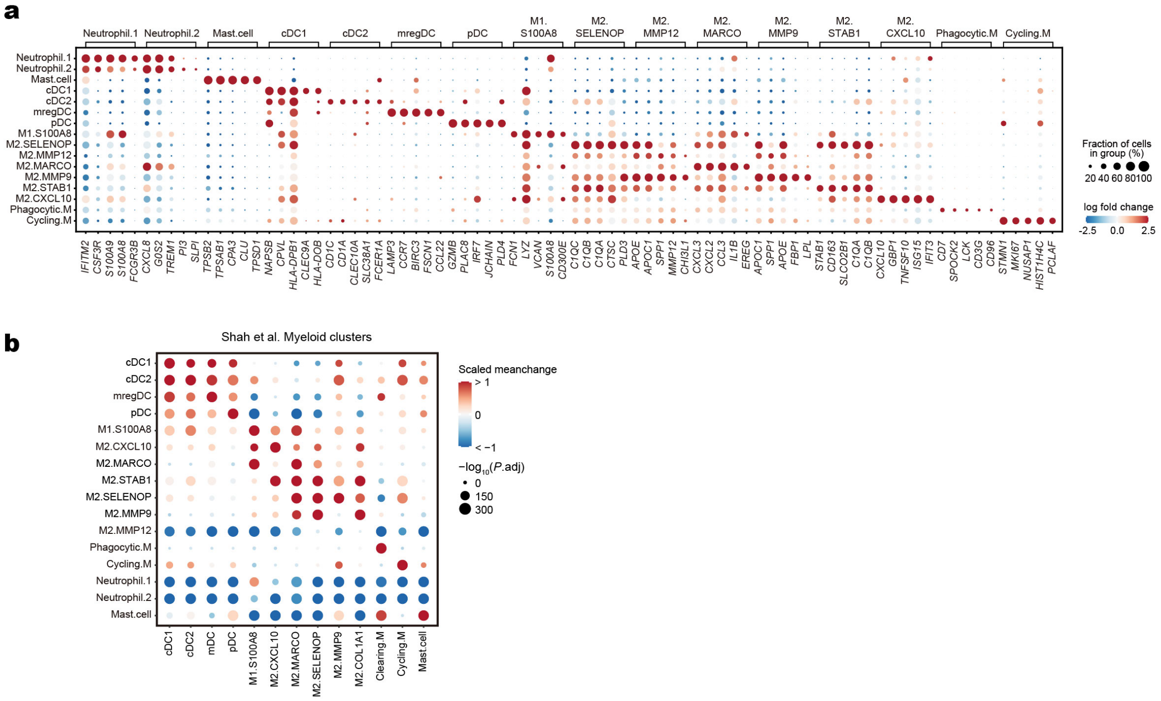

Supplementary Fig. 15 | Marker gene expression of myeloid cell phenotypes. a, Dot plot of scaled marker gene expression (averaged per cluster) for coarse-grained myeloid cell subclusters, showing differentially expressed genes in columns and clusters in rows. Genes are grouped by cluster and five genes per cluster that are highlighted. A color gradient represents the mean expression within each of the subcluster of myeloid cells, while the sizes of the depicted point indicates the fraction of cells in the categories expressing a gene. **b**, Dot plot showing comparison of myeloid cell subclusters (this study) and published myeloid cell subclusters using AUCell algorithm to verify the cell annotation. A color gradient, transitioning from red (representing high scaled mean change) to blue (representing high scaled mean change), encodes the Z-scaled scores, while the sizes of the depicted points are governed by the Benjamini-Hochberg-adjusted -$\log_{10}$(P values).

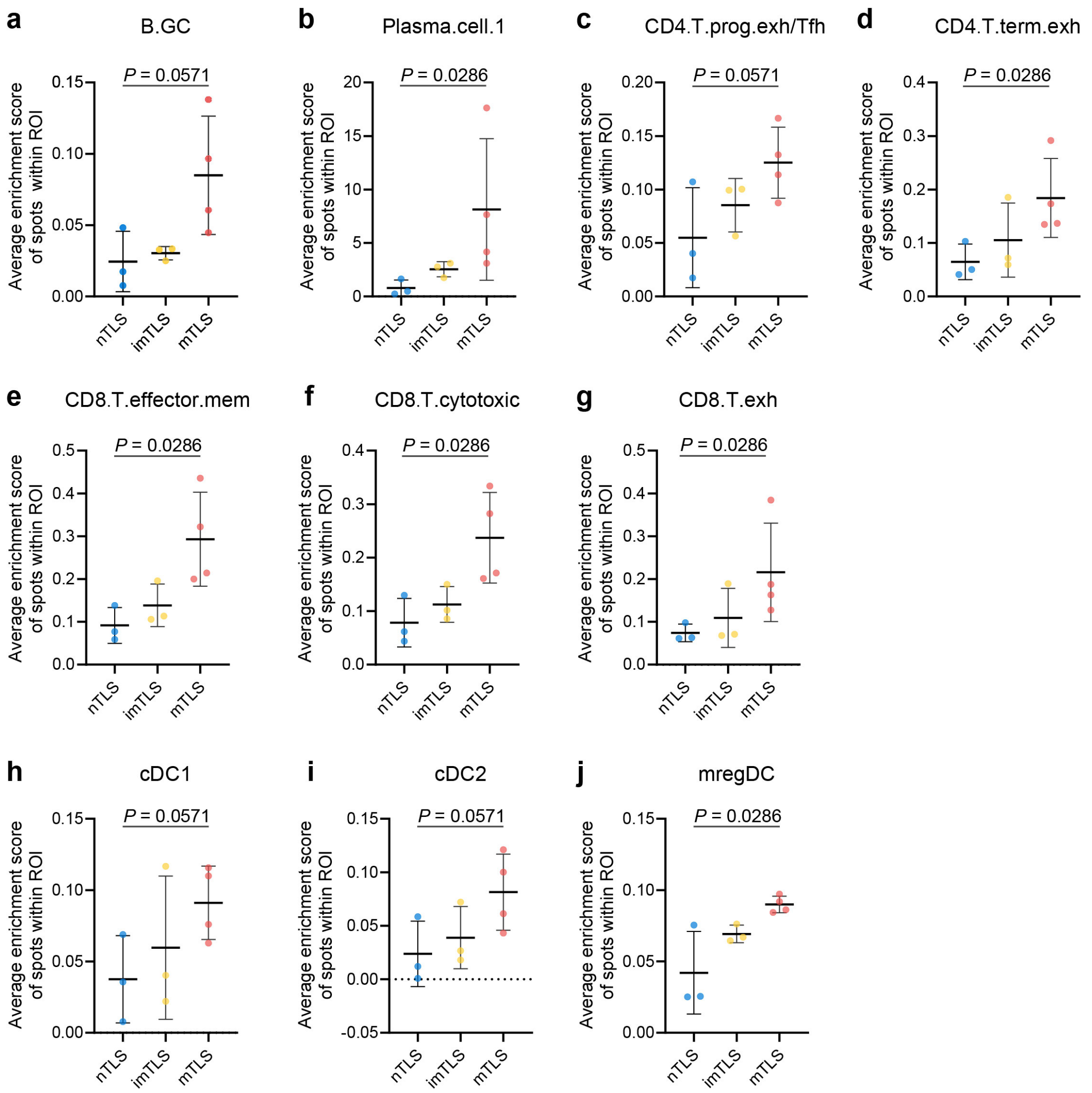

Supplementary Fig. 16 | Average enrichment score by spatial transcriptomics in HNSCC. a-j, Average enrichment score of B.GC **(a)**, plasma.cell.1 **(b)**, CD4.T.prog.exh **(c)**, CD4.T.term.exh **(d)**, CD8.T.effector.mem **(e)**, CD8.T.cytotoxic **(f)**, CD8.T.exh **(g)**, cDC1 **(h)**, cDC2 **(i)**, mregDC **(j)** in spots within the region of interest (ROI) for TLS across different TLS statuses (nTLS, imTLS or mTLS) detected by spatial transcriptomics (n = 3 independent samples nTLS, n = 3 independent samples imTLS, n = 4 independent samples mTLS, one-tailed Mann-Whitney *U*-test; for dot plots: center line, mean; whiskers, standard deviation). ROI, Region of interest.

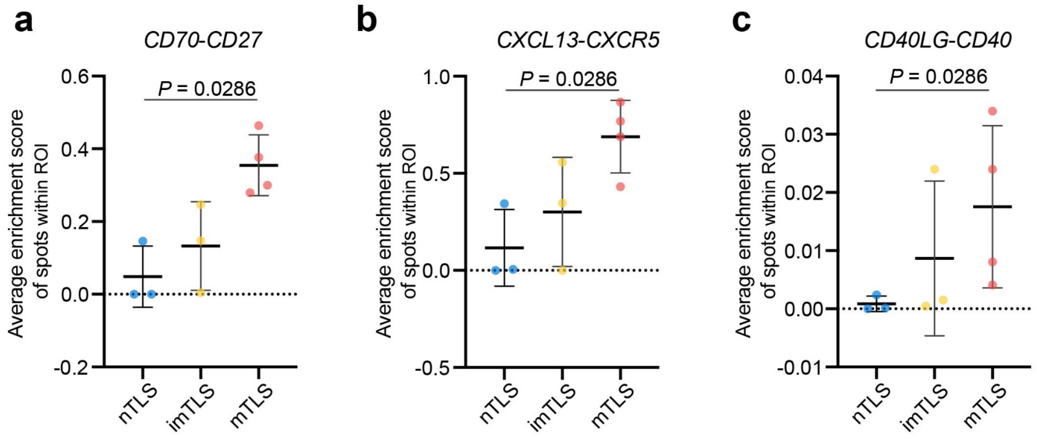

Supplementary Fig. 17 | Statistics on cell–cell communication and receptor–ligand pairs in HNSCC. a-c, Average enrichment score of *CD70–CD27* **(f)**, *CXCL13–CXCR5* **(g)**, and *CD40LG–CD40* **(h)** spots within the region of interest (ROI) for TLS across different TLS statuses detected by spatial transcriptomics (n = 3 independent samples nTLS, n = 3 independent samples imTLS, n = 4 independent samples mTLS, one-tailed Mann-Whitney *U*-test; for dot plots: center line, mean; whiskers, standard deviation). ROI, Region of interest.

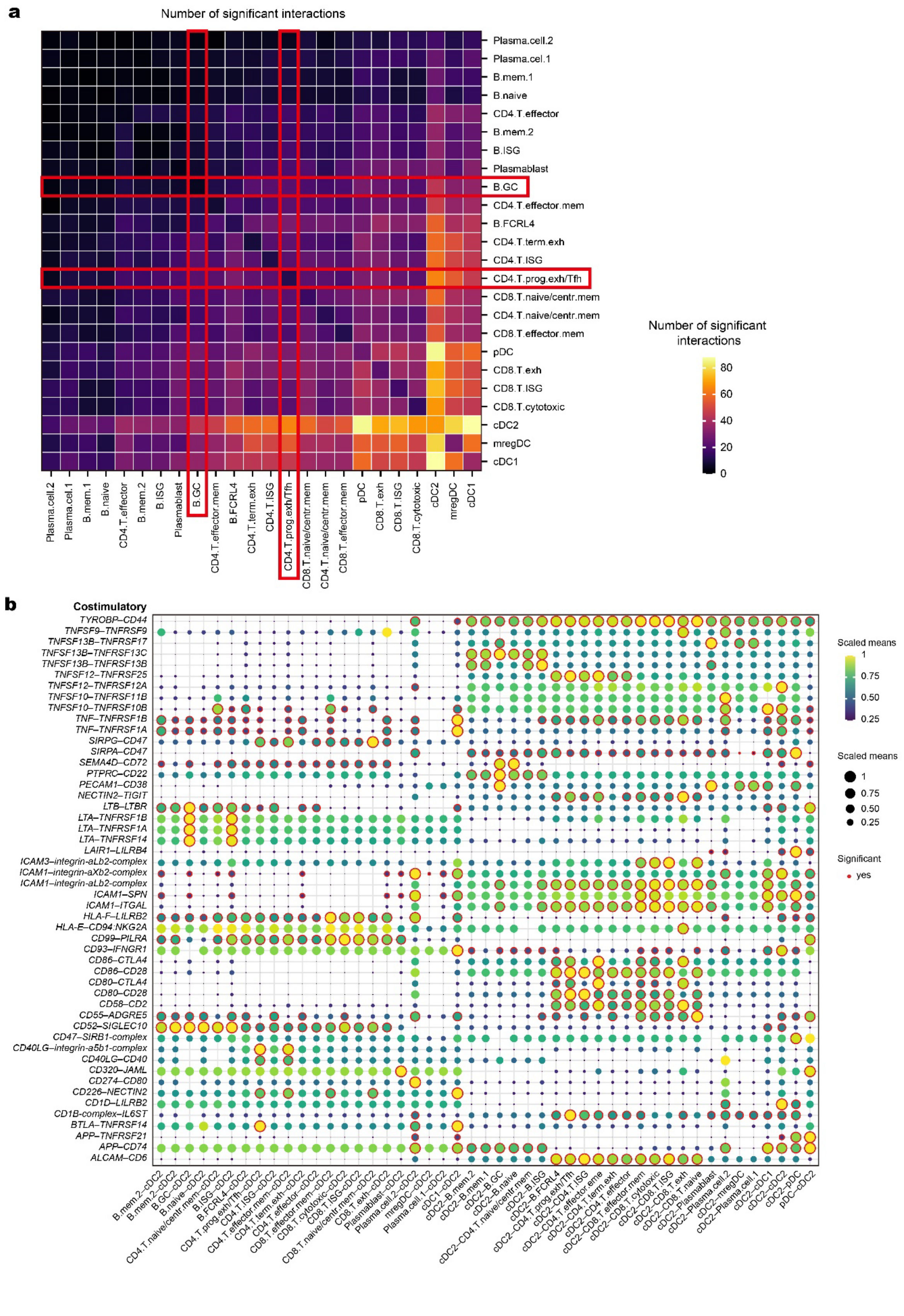

Supplementary Fig. 18 | Cell-cell significant interaction information of HNSCC. a, Heatmaps depicting cell–cell communications in immune costimulatory quantified by CellPhoneDB in all HNSCC samples. **b**, Bubble

heatmap of the mean interaction strength for selected ligand–receptor pairs in various immune cell clusters. Both dot size and color indicate scaled mean expression of selected ligand–receptor pairs. The red circle represents a statistically significance (*P* value < 0.05) using CellphoneDB statistical analysis method.

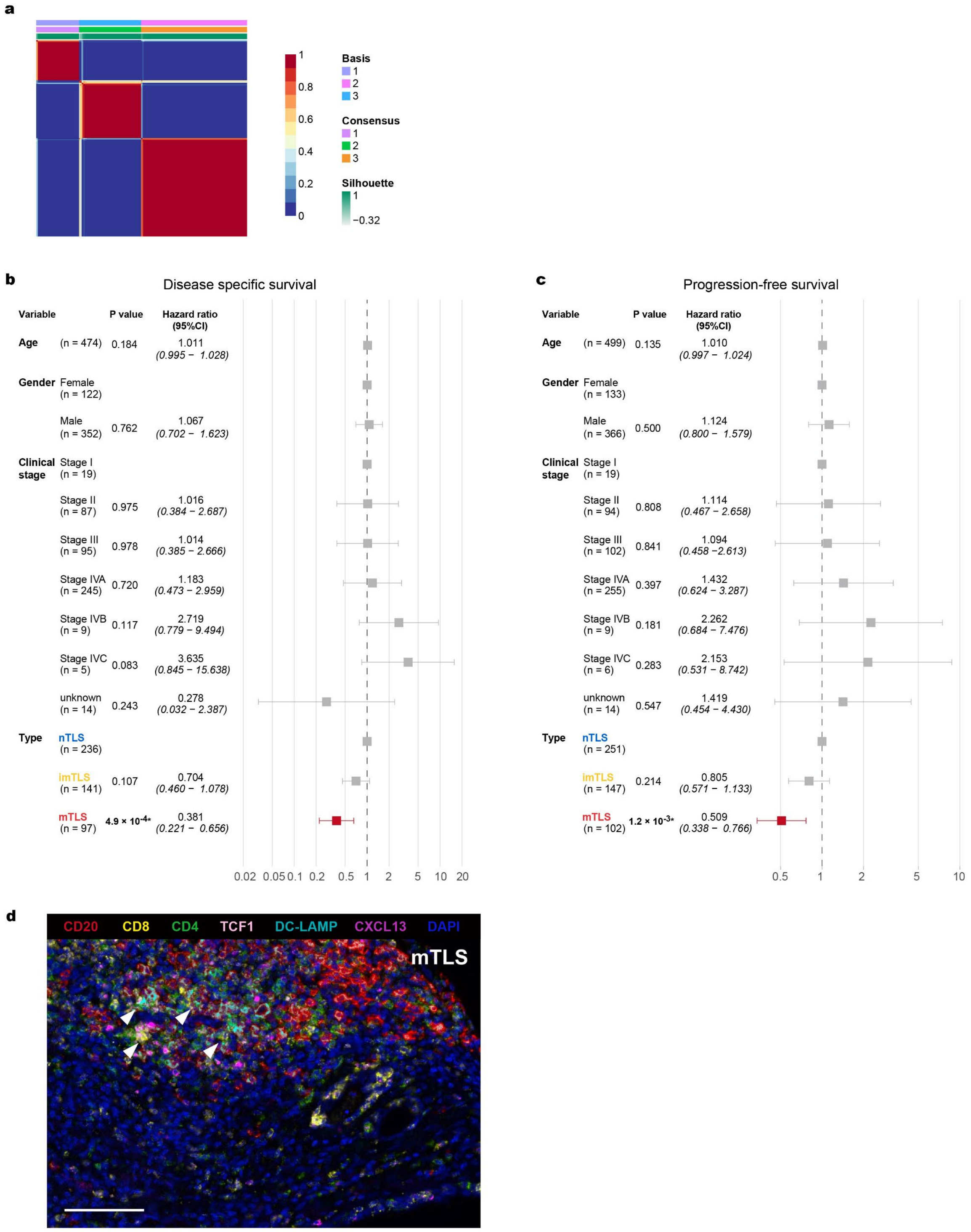

Supplementary Fig. 19 | Multivariate Cox regression model analysis of TLS groupings in HNSCC. a, Heatmap showing the results of non-negative matrix factorization (NMF) clustering applied to the data of cell type abundance in patients from TCGA-HNSC. **b,** Multivariate Cox proportional regression for disease specific survival (DSS), with clinical variables. For every variable included in the analysis, the reference level is the first one. A grey bar symbolizes a *P* value > 0.05; and blue and red bars symbolizes *P* value < 0.05 positively and

negatively, respectively. Error bars represent the 95% confidence interval. **c,** Multivariate Cox proportional regression for progression free interval (PFI), with clinical variables. For every variable included in the analysis, the reference level is the first one. A grey bar symbolizes a *P* value > 0.05; and blue and red bars symbolizes *P* value < 0.05 positively and negatively, respectively. Error bars represent the 95% confidence interval. **d,** Representative images of a mIHC-stained different subclusters of $CD4^+$ Tex^{prog} cells in HNSCC tumor in mTLS status (Data were repeated 20 times independently with similar results). Multiplex immunofluorescence assay of CD20 (red), CD8 (yellow), CD4 (green), TCF1 (pink), DC-LAMP (cyan), CXCL13 (magenta) and DAPI (blue). Scale bars = 50 μm.

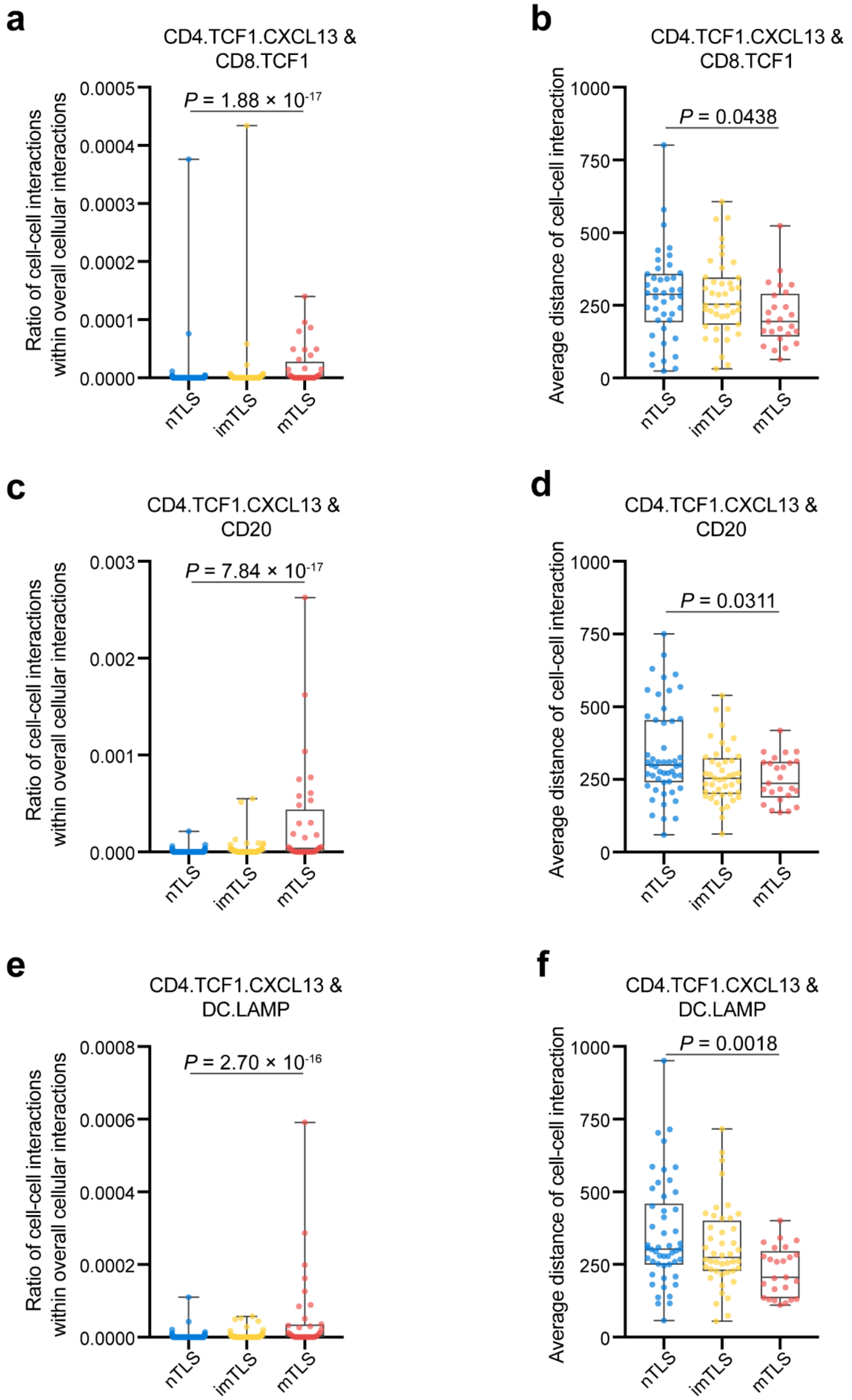

Supplementary Fig. 20 | Statistic of the ratio of cell-cell interactions and the average distance of cell-cell interactions. a-f, Ratio of cell-cell interactions within overall cellular interactions and average distance of cell-

cell interactions between $CD4^{+}TCF1^{+}CXCL13^{+}$ cells and $CD8^{+}TCF1^{+}$ cells **(a, b),** $CD20^{+}$ cells **(c, d)**, and $LAMP^{+}$ DCs **(e, f)** within overall cellular interactions in HNSCC tissue with different TLS statuses. Sample sizes for **a, c, e** are as follows: n = 303 independent samples nTLS, n = 83 independent samples imTLS, n = 36 independent samples mTLS. Sample sizes for b are as follows: n = 42 independent samples nTLS, n = 42 independent samples imTLS, n = 25 independent samples mTLS; sample sizes for d are as follows: n = 51 independent samples nTLS, n = 44 independent samples imTLS, n = 25 independent samples mTLS; sample sizes for f are as follows: n = 50 independent samples nTLS, n = 44 independent samples imTLS, n = 25 independent samples mTLS. Statistical significance was determined using a two-tailed Mann-Whitney *U*-test. For box plots: box center line, median; box limits, upper and lower quartiles; box whiskers, maximum and minimum values.

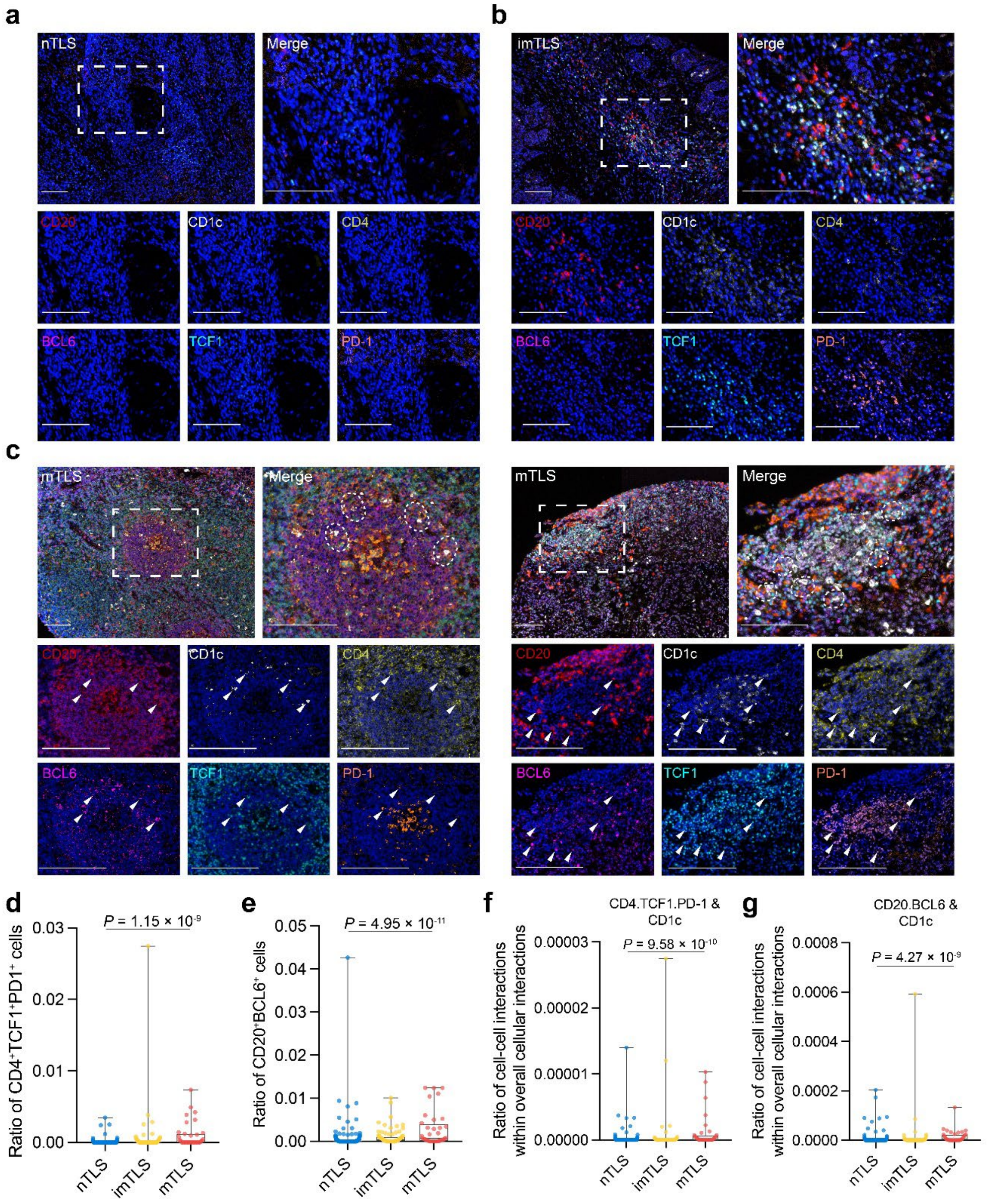

Supplementary Fig. 21 | Representative images and quantification of a mIHC-stained different subclusters of BCL6^{+}CD20^{+} B cells, CD4^{+}TCF1^{+}PD-1^{+} Tex^{prog} cells in HNSCC tumor with different TLS status. a-c Images of the mIHC-stained HNSCC tissues with different TLS status showing co-localization of B cell (CD20, BCL6), CD4^{+} Tex^{prog} (CD4, PD-1 and TCF1), and cDC2s (CD1c) in HNSCC across different TLS statuses. nTLS were repeated 286 times independently with similar results, imTLS were repeated 83 times independently with similar results, mTLS were repeated 36 times independently with similar results. Scale bars = 100 μm. **d, e**, Ratio of CD4^{+}TCF1^{+}PD-1^{+} cells **(d)** and CD20^{+}BCL6^{+} cells **(e)** in total cells of HNSCC tissue with different TLS

statuses (n = 286 independent samples nTLS, n = 83 independent samples imTLS, n = 36 independent samples mTLS), respectively. f, g, Ratio of CD4$^+$ TCF1$^+$PD-1$^+$ cells and CD1c$^+$ cells **(f)**, CD20$^+$BCL6$^+$ cells and CD1c$^+$ cells **(g)** interactions within overall cellular interactions of HNSCC tissue with different TLS statuses (n = 286 independent samples nTLS, n = 83 independent samples imTLS, n = 36 independent samples mTLS), respectively. (two-tailed Mann-Whitney *U*-test; for box plots: box center line, median; box limits, upper and lower quartiles; box whiskers, maximum and minimum values).

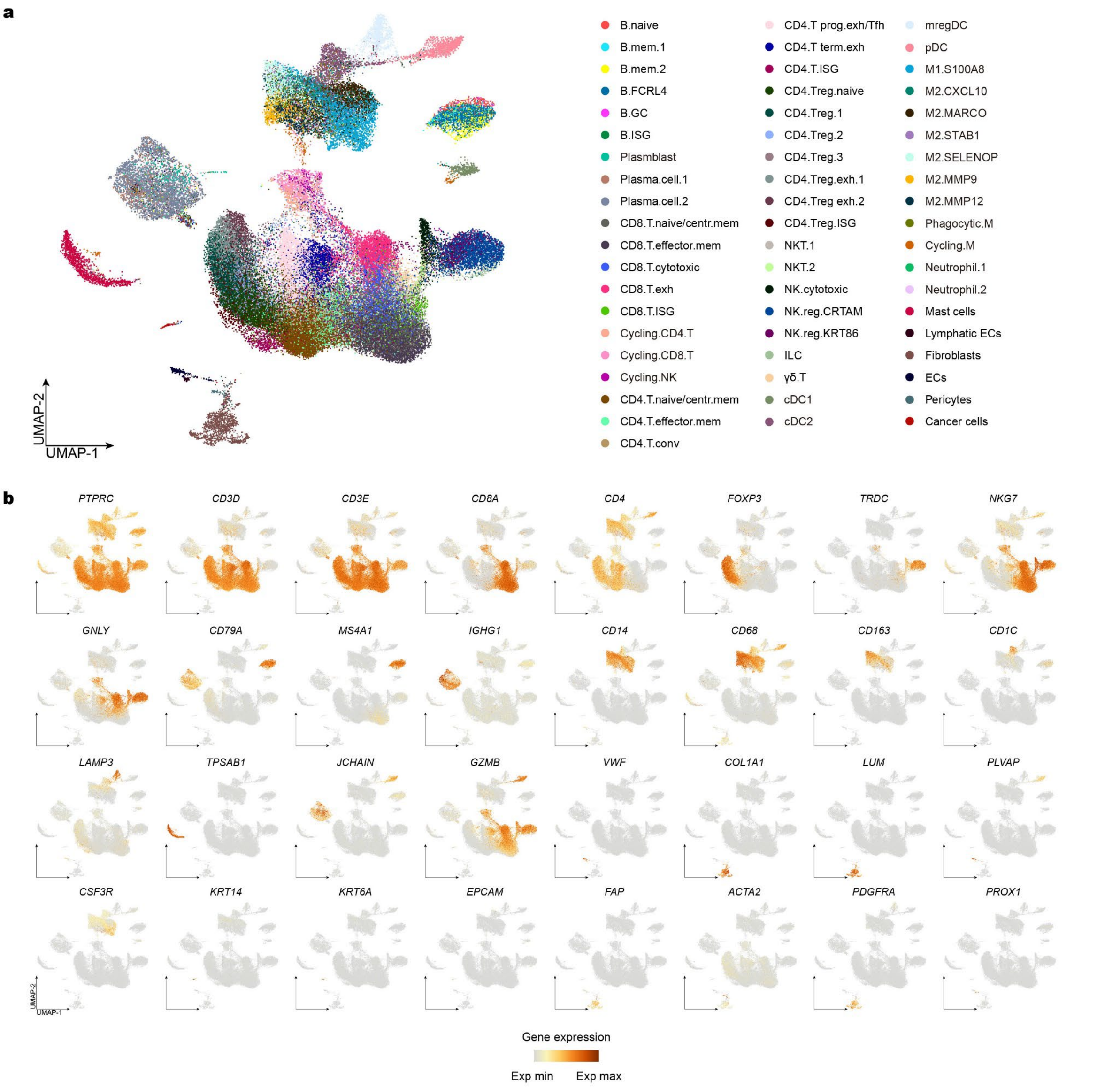

Supplementary Fig. 22 | Consistent annotation transfer from our scRNA-seq data to query scRNA-seq data. **a,** UMAP plot of 80,794 cells profiled by all scRNA-seq from GSE200996, colored by cell types. TOSICA was used to annotate cell types from our scRNA-seq data to the published dataset (GSE200996). The 11 broad cellular lineages annotation containing ECs, lymphatic ECs, stromal cells (pericytes, fibroblasts), lymphoid cells (T/NK cells, B cells, plasma cells), myeloid cells, neutrophils and cancer cells were as same as our scRNA-seq data. The annotation of the 11 broad cellular lineages included ECs, lymphatic ECs, stromal cells (pericytes, fibroblasts), lymphoid cells (T/NK cells, B cells, and plasma cells), myeloid cells, neutrophils, and cancer cells was the same as our scRNA-seq data. **b,** UMAP plot of normalized expression of specific markers.
