# Supplementary material for: Mature tertiary lymphoid structures evoke intra-tumoral T and B cell responses via progenitor exhausted CD4+ T cells in head and neck cancer
Source: Nat Commun. 2025 May 7;16:4228. doi: 10.1038/s41467-025-59341-w (PMC12059173; doi:10.1038/s41467-025-59341-w)
Supplement: Supplementary file 2 — Description of Additional Supplementary Files [file 41467_2025_59341_MOESM2_ESM.pdf]

Description of Additional Supplementary Files

Supplementary Data 1.
Clinical overview of the patient cohort comprising 14 HNSCC samples.

Supplementary Data 2.
Clinical overview of the patient cohort comprising 422 HNSCC samples.

Supplementary Data 3.
Cell type and cell subtype markers. Different clusters were annotated on the basis of top 50 marker genes identified in differential gene expression analysis of scRNA-seq data.

Supplementary Data 4.
The interaction data for ligand–receptor pairs in various immune cell clusters.

Supplementary Data 5.
Metadata information, TLS status and the quantitative analysis of B cells, T cells, DC and chemokine in TMAs comprising 422 HNSCC samples.

Supplementary Data 6.
The antibodies used for IHC and mIHC in this study.
