# Supplementary material for: Mature tertiary lymphoid structures evoke intra-tumoral T and B cell responses via progenitor exhausted CD4+ T cells in head and neck cancer
Source: Nat Commun. 2025 May 7;16:4228. doi: 10.1038/s41467-025-59341-w (PMC12059173; doi:10.1038/s41467-025-59341-w)
Supplement: Supplementary file 9 — Reporting Summary [file 41467_2025_59341_MOESM9_ESM.pdf]

nature portfolio

Corresponding author(s): Zhi-Jun Sun, Wei-Wei Deng

Last updated by author(s): Mar 16, 2025

# Reporting Summary

Nature Portfolio wishes to improve the reproducibility of the work that we publish. This form provides structure for consistency and transparency in reporting. For further information on Nature Portfolio policies, see our Editorial Policies and the Editorial Policy Checklist.

## Statistics

For all statistical analyses, confirm that the following items are present in the figure legend, table legend, main text, or Methods section.

| n/a | Confirmed | |
|---|---|---|
| ☐ | ☒ | The exact sample size (*n*) for each experimental group/condition, given as a discrete number and unit of measurement |
| ☐ | ☒ | A statement on whether measurements were taken from distinct samples or whether the same sample was measured repeatedly |
| ☐ | ☒ | The statistical test(s) used AND whether they are one- or two-sided<br>*Only common tests should be described solely by name; describe more complex techniques in the Methods section.* |
| ☒ | ☐ | A description of all covariates tested |
| ☐ | ☒ | A description of any assumptions or corrections, such as tests of normality and adjustment for multiple comparisons |
| ☐ | ☒ | A full description of the statistical parameters including central tendency (e.g. means) or other basic estimates (e.g. regression coefficient) AND variation (e.g. standard deviation) or associated estimates of uncertainty (e.g. confidence intervals) |
| ☐ | ☒ | For null hypothesis testing, the test statistic (e.g. *F*, *t*, *r*) with confidence intervals, effect sizes, degrees of freedom and *P* value noted<br>*Give P values as exact values whenever suitable.* |
| ☒ | ☐ | For Bayesian analysis, information on the choice of priors and Markov chain Monte Carlo settings |
| ☒ | ☐ | For hierarchical and complex designs, identification of the appropriate level for tests and full reporting of outcomes |
| ☐ | ☒ | Estimates of effect sizes (e.g. Cohen's *d*, Pearson's *r*), indicating how they were calculated |

*Our web collection on statistics for biologists contains articles on many of the points above.*

## Software and code

Policy information about availability of computer code

| | |
|---|---|
| Data collection | CeleScope v1.13.0 was used for scRNA-seq and scTCR/BCR-seq data. Space Ranger (v1.0.0) was used for spatial transcriptomic data. Vectra v3.0.7 was used for scanning mIHC slides. 3DHISTECH v1.0.8 was used for scanning HE and IHC slides. |
| Data analysis | All scRNA-seq data were processed using CeleScope (v1.13.0), Python (v3.8, v3.9), Scanpy (v1.10.3), Anndata (v0.10.8), Umap-learn (0.5.5), Numpy (1.24.3), Scrublet (v0.2.3), Harmony (v0.0.9), CellphoneDB (v4.1.0), Bulk2Space (version 1.0.0), TOSICA (version 1.0.0), Augur (version 1.0.0) and decoupleR (v1.5.0).<br>All scTCR/BCR-seq data were processed using CeleScope (v1.13.0), Python (v3.8, v3.9), Scanpy (v1.10.3), and Scirpy (v0.17.2).<br>All spatial transcriptomic data were processed using Space Ranger (v1.0.0), Python (v3.8, v3.9), Scanpy (v1.10.3) and Cell2location (v0.1.3).<br>All mIHC data were processed using inForm software (v2.4).<br><br>The software was used for data statistics and plotting, as listed below:<br>GraphPad Prism v9.0,<br>R v4.4.2,<br>Seurat v4.3.0,<br>dplyr v1.1.3,<br>tidyr v1.3.0,<br>ggplot2 v.3.4.3,<br>scales v1.2.1,<br>stringr v1.5.0,<br>ggrastr v1.0.2, |

tidyverse v2.0.0
openxlsx v4.2.5.2
pheatmap v1.0.12
forcats v1.0.0
RColorBrewer v1.1-3
emmeans v1.8.7
cowplot v1.1.1
Immcantation v4.5.0

For manuscripts utilizing custom algorithms or software that are central to the research but not yet described in published literature, software must be made available to editors and reviewers. We strongly encourage code deposition in a community repository (e.g. GitHub). See the Nature Portfolio guidelines for submitting code & software for further information.

## Data

Policy information about availability of data

All manuscripts must include a data availability statement. This statement should provide the following information, where applicable:

- Accession codes, unique identifiers, or web links for publicly available datasets
- A description of any restrictions on data availability
- For clinical datasets or third party data, please ensure that the statement adheres to our policy

The data deposited and made public are compliant with the regulations of the Ministry of Science and Technology of China (confirmation number: 2024BAT01131). The raw scRNA-seq, scTCR/BCR-seq and spatial transcriptomic data reported in this paper have been deposited at the Genome Sequence Archive at the National Genomics Data Center (China) under the BioProject (PRJCA025983). Since these data are related to human genetic resources, the raw data can be obtained directly by requesting and following the GSA guidelines for academic use at the website (https://ngdc.cncb.ac.cn/bioproject/browse/PRJCA025983) after the user logs in to the GSA database using their academic institution email address. The request will be responded to within two weeks. Once access is granted, users have six months to download the data.

The processed, publicly available scRNA-seq and spatial transcriptomic matrices used in this study are also available on Figshare. The scRNA-seq data for whole tumor cells (https://doi.org/10.6084/m9.figshare.26044012.v1), the paired scRNA-seq and scTCR/BCR-seq data for CD45+ cells (https://doi.org/10.6084/m9.figshare.26044039.v1), and the spatial transcriptomic data (https://doi.org/10.6084/m9.figshare.26044042.v1) are all accessible on Figshare.

Other public data used in this study include RNA-seq data from TCGA datasets (https://portal.gdc.cancer.gov/), scRNA-seq data (GSE200996) and gene expression data (GSE93157) from GEO datasets (https://www.ncbi.nlm.nih.gov/geo/).The data generated in this study are available and provided in the Source Data file.

## Research involving human participants, their data, or biological material

Policy information about studies with human participants or human data. See also policy information about sex, gender (identity/presentation), and sexual orientation and race, ethnicity and racism.

| | |
|---|---|
| Reporting on sex and gender | 1. 4 out of 14 HNSCC patients are female for scRNA-seq in this study, no sex or gender analysis was carried out.<br>2. 88 out of 422 HNSCC patients are female in validation cohort, no sex or gender analysis was carried out.<br>Gender analysis was not performed because we focused on the differences between TLS status instead of genders. |
| Reporting on race, ethnicity, or other socially relevant groupings | The research was not involved in race, ethnicity or other socially relevant groupings. The relevant information was not take under consideration. |
| Population characteristics | 1. The scRNA-seq cohort of 14 HNSCC patients were patients with primary HNSCC and samples were collected between Sep 2022 and Jun 2023. They were treatment-naive (primary). Their ages range from 31 to 82, with 10 male patients and 4 female patients. The clinical information is summarized in Supplementary Data 1.<br>2. The validation cohortl of 422 HNSCC patients includes 76 patients with Grade I stage, 280 patients with Grade II stage and 66 patients with Grade III stage HNSCC. They were treatment-naive. Their ages range from 21 to 88, with 334 male patients and 88 female patients. The clinical information is summarized in Supplementary Data2. |
| Recruitment | Our study does not involve patient recruitment. |
| Ethics oversight | This study was approved by the Medical Ethics Committee of the School and Hospital of Stomatology, Wuhan University. Written informed consent was obtained from each patient. HNSCC samples were derived from the Department of Oral and Maxillofacial Surgery, Wuhan University School and Hospital of Stomatology. |

Note that full information on the approval of the study protocol must also be provided in the manuscript.

# Field-specific reporting

Please select the one below that is the best fit for your research. If you are not sure, read the appropriate sections before making your selection.

[x] Life sciences [ ] Behavioural & social sciences [ ] Ecological, evolutionary & environmental sciences

For a reference copy of the document with all sections, see nature.com/documents/nr-reporting-summary-flat.pdf

# Life sciences study design

All studies must disclose on these points even when the disclosure is negative.

| | |
|---|---|
| Sample size | Cohort for scRNA-seq, scTCR/BCR-seq and spatial transcriptomics: n=14, including different TLS status; Cohort for spatial transcriptomics: n=422, including different TLS status. Since samples used for scRNA-seq were human subjects, we collected as many samples as possible within our timeframe.<br>Details regarding sample size of all experiments were provided in the figure captions. We followed the routine biological replicate requirement in experiment section, n >= 3 for each group. |
| Data exclusions | For sequencing data, we excluded low-quality cells if abnormalities exist in (1) cell library sizes, (2) the numbers of expressed genes; (3) theproportion of mitochondrial gene counts. The details of cut-off line could be checked in Methods. |
| Replication | All replications were successful, and the detailed information was provided in corresponding figure legends. |
| Randomization | This study relied on the use of human samples with no interventions performed on the human subjects. Randomization was not applicable. |
| Blinding | The TLS status of tumor was known before scRNA-seq and spatial transcriptomics. There is no intervention to patients recruited, and it's not a clinical trail. Blinding is not applicable. For remaining experiments in our study, no binding was used. |

# Reporting for specific materials, systems and methods

We require information from authors about some types of materials, experimental systems and methods used in many studies. Here, indicate whether each material, system or method listed is relevant to your study. If you are not sure if a list item applies to your research, read the appropriate section before selecting a response.

## Materials & experimental systems

| n/a | Involved in the study | |
|---|---|---|
| ☐ | ☒ | Antibodies |
| ☒ | ☐ | Eukaryotic cell lines |
| ☒ | ☐ | Palaeontology and archaeology |
| ☒ | ☐ | Animals and other organisms |
| ☒ | ☐ | Clinical data |
| ☒ | ☐ | Dual use research of concern |
| ☒ | ☐ | Plants |

## Methods

| n/a | Involved in the study | |
|---|---|---|
| ☒ | ☐ | ChIP-seq |
| ☐ | ☒ | Flow cytometry |
| ☒ | ☐ | MRI-based neuroimaging |

## Antibodies

Antibodies used

The following primary antibodies were used for immunohistochemistry. They are listed as antigen first, followed by dilutions, supplier, catalog number as applicable.
1) anti-CD20, 1:200, Cell Signaling Technology, Cat#48750;
2) anti-CD3ε, 1:150, Abcam, Cat#ab16669;
3) anti-CD23, 1:200, Abcam, Cat#ab92495.

The following primary antibodies were used for immunohistochemistry by OCT embedding. They are listed as antigen first, followed by dilutions, supplier, catalog number as applicable.
1) CD20 Monoclonal Antibody, 1:200, eBioscience, Clone 2H7, Cat#14-0209-82;
2) CD3 Monoclonal Antibody, 1:200, eBioscience, Clone UCHT1, Cat#14-0038-82;
3) CD23 Monoclonal Antibody, 1:200, eBioscience, Clone EBVCS2, Cat#14-0238-82.

The following primary antibodies were used for multiplex immunohistochemistry. They are listed as antigen first, followed by dilutions, supplier, catalog number as applicable.
1) anti-CD20, 1:1000, Cell Signaling Technology, Cat#48750;
2) anti-TCF1, 1:600, Cell Signaling Technology, Cat#2203;
3) anti-CD4, 1:1000, Cell Signaling Technology, Cat#48274;
4) anti-CD8, 1:800, Cell Signaling Technology, Cat#85336;
5) anti-PD-1, 1:600, Cell Signaling Technology, Cat#43248;
6) anti-Granzyme B, 1:800, Cell Signaling Technology, Cat#46890;
7) anti-CCR7, 1:1200, Abcam, Cat#ab253187;
8) anti-CXCR5, 1:1200, Abcam, Cat#ab46218;
9) anti-DC-LAMP, 1:600, Atlas Antibodies, Cat#HPA051467;
10) anti-CXCL13, 1:600, Abcam, Cat#ab92495;
11) anti-CD23, 1:900, Abcam, Cat#ab92495;
12) anti-BCL6, 1:600, Cell Signaling Technology, Cat#89369;

13) anti-CD1c, 1:1400, Abcam, Cat#ab246520.

The following primary antibodies were used for flow cytometry and magnetic-activated cell sorting. They are listed as antigen first, followed by supplier, catalog number as applicable.
1) Anti-human CD45, Biolegend, APC/Cyanine7, Clone 2D1, Cat#368515;
2) CD45 (TIL) MicroBeads human, Miltenyi Biotec, Cat#130-118-780.

Validation

No customized antibodies were used. Validation data of the antibodies purchased from commercial vendors are available on the manufactures' website and datasheets.
1) anti-CD20, Cell Signaling Technology, Cat#48750;
https://www.cellsignal.cn/products/primary-antibodies/cd20-e7b7t-xp-rabbit-mab/48750
2) anti-CD3ε Abcam, Cat#ab16669;
https://www.abcam.cn/products/primary-antibodies/cd3-epsilon-antibody-sp7-ab16669.html
3) anti-CD23, Abcam, Cat#ab92495;
https://www.abcam.cn/products/primary-antibodies/cd23-antibody-epr3617-ab92495.html
4) CD20 Monoclonal Antibody, eBioscience, Clone 2H7, Cat#14-0209-82;
https://www.thermofisher.cn/cn/zh/antibody/product/CD20-Antibody-clone-2H7-Monoclonal/14-0209-82
5) CD3 Monoclonal Antibody, eBioscience, Clone UCHT1, Cat#14-0038-82
https://www.thermofisher.cn/cn/zh/antibody/product/CD3-Antibody-clone-UCHT1-Monoclonal/14-0038-82
6) CD23 Monoclonal Antibody, eBioscience, Clone EBVCS2, Cat#14-0238-82
https://www.thermofisher.cn/cn/zh/antibody/product/CD23-Antibody-clone-EBVCS2-Monoclonal/14-0238-82
7) anti-CD20, Cell Signaling Technology, Cat#48750;
https://www.cellsignal.cn/products/primary-antibodies/cd20-e7b7t-xp-rabbit-mab/48750
8) anti-TCF1, Cell Signaling Technology, Cat#2203;
https://www.cellsignal.cn/products/primary-antibodies/tcf1-tcf7-c63d9-rabbit-mab/2203
9) anti-CD4, Cell Signaling Technology, Cat#48274;
https://www.cellsignal.cn/products/primary-antibodies/cd4-ep204-rabbit-mab/48274
10) anti-CD8, Cell Signaling Technology, Cat#85336;
https://www.cellsignal.cn/products/primary-antibodies/cd8a-d8a8y-rabbit-mab/85336
11) anti-PD-1, Cell Signaling Technology, Cat#43248;
https://www.cellsignal.cn/products/primary-antibodies/pd-1-eh33-mouse-mab/43248
12) anti-Granzyme B, Cell Signaling Technology, Cat#46890;
https://www.cellsignal.cn/products/primary-antibodies/granzyme-b-d6e9w-rabbit-mab/46890
13) anti-CCR7, Abcam, Cat#ab253187;
https://www.abcam.cn/products/primary-antibodies/ccr7-antibody-epr23192-57-ab253187.html
14) anti-CXCR5, Abcam, Cat#ab46218;
https://www.abcam.cn/products/primary-antibodies/cxcr5-antibody-ab46218.html
15) anti-DC-LAMP, Atlas Antibodies, Cat#HPA051467;
https://www.atlasantibodies.com/products/primary-antibodies/triple-a-polyclonals/anti-lamp3-antibody-hpa051467/
16) anti-CXCL13, Abcam, Cat#ab246518;
https://www.abcam.cn/products/primary-antibodies/cxcl13-antibody-epr23400-92-ab246518.html
17) anti-CD23, Abcam, Cat#ab92495;
https://www.abcam.cn/products/primary-antibodies/cd23-antibody-epr3617-ab92495.html
18) anti-BCL6, Cell Signaling Technology, Cat#89369;
https://www.cellsignal.cn/products/primary-antibodies/bcl6-e5i8i-rabbit-mab/89369
19) anti-CD1c, Abcam, Cat#ab246520.
https://www.abcam.cn/products/primary-antibodies/cd1c-antibody-epr23189-196-ab246520.html
20) Anti-human CD45, Biolegend, APC/Cyanine7, Clone 2D1, Cat#368515;
https://www.biolegend.com/en-gb/products/apc-cyanine7-anti-human-cd45-antibody-12400
21) CD45 (TIL) MicroBeads human, Miltenyi Biotec, Cat#130-118-780;
https://www.miltenyibiotec.com/UN-en/products/cd45-til-microbeads-human.html#130-118-780

## Plants

Seed stocks

Not applicable.

Novel plant genotypes

Not applicable.

Authentication

Not applicable.

# Flow Cytometry

## Plots

Confirm that:

- [x] The axis labels state the marker and fluorochrome used (e.g. CD4-FITC).
- [x] The axis scales are clearly visible. Include numbers along axes only for bottom left plot of group (a 'group' is an analysis of identical markers).
- [x] All plots are contour plots with outliers or pseudocolor plots.
- [x] A numerical value for number of cells or percentage (with statistics) is provided.

## Methodology

| | |
|---|---|
| Sample preparation | Tumors were collected from mice and made into cell suspensions by gentleMACS dissociator and digestive enzyme (Miltenyi Biotec) according to the manufacturer's instructions. Then, the samples were passed through 200-mesh nylon mesh filters to obtain single-cell suspensions. |
| Instrument | CytoFLEX flow cytometer (Beckman) |
| Software | CytExpert (v2.4) used for data collection, Flowlo (v10.6.2) used for data analysis. |
| Cell population abundance | No fluorescence-activated cell sorting was performed. |
| Gating strategy | To analyze the proportion of HNSCC patients, cells were gated as follows.<br>1.First gated based on physical parameters SSC-A/FSC-A.<br>2.FVD-CD45+ for general immune cells.<br>Detailed information is available in the Extended Data Figure 3. |

- [x] Tick this box to confirm that a figure exemplifying the gating strategy is provided in the Supplementary Information.
